# Scale Dependence of Woody Plant β‐Diversity in a Tropical Rainforest Metacommunity

**DOI:** 10.1002/ece3.74022

**Published:** 2026-07-12

**Authors:** Jie Yao, Xinran Li, Shinichi Tatsumi, Yi Ding, Jihong Huang, Yue Xu, Zhidong Zhang, Wenxing Long, Runguo Zang

**Affiliations:** ^1^ Ecology and Nature Conservation Institute, Chinese Academy of Forestry; Key Laboratory of National Forestry and Grassland Administration on Forest Ecosystem Conservation and Restoration Beijing China; ^2^ CAS Key Laboratory of Forest Ecology and Silviculture, Institute of Applied Ecology Chinese Academy of Sciences Shenyang China; ^3^ Bawangling Observation and Research Station of National Park of Hainan Tropical Rainforest Changjiang China; ^4^ Graduate School of Agriculture Kyoto University Kyoto Japan; ^5^ Hebei Provincial Key Laboratory of Forest Trees Germplasm Resources and Forest Protection, College of Forestry Hebei Agricultural University Baoding China; ^6^ Wuzhishan National Long‐Term Forest Ecosystem Monitoring Research Station, Hainan Key Laboratory for Sustainable Utilization of Tropical Bioresource, College of Forestry Hainan University Haikou China; ^7^ Institute of Hainan National Park Haikou China

**Keywords:** environmental heterogeneity, metacommunity, spatial scale, spatial structure, species replacement and richness difference, β‐Diversity

## Abstract

β‐diversity is a central concept to understanding community assembly, yet how its decomposition components and underlying drivers vary with spatial extent remains insufficiently understood. We used a nested metacommunity framework to examine the scale dependence of total β‐diversity and its species replacement and richness difference components, and to identify how environmental and spatial predictors contribute across scales. We analyzed woody plant diversity in 250 plots arranged on a contiguous 1 × 1 km grid in a tropical rainforest on Hainan, China. By aggregating nearest‐neighbor plots into metacommunities, we constructed a continuous gradient of spatial extent and quantified total β‐diversity (BDtotal), species replacement (Repl), and richness difference (RichDif) for each metacommunity realization. β‐diversity increased modestly with spatial extent, but its internal structure was remarkably stable: species replacement consistently dominated total β‐diversity, whereas richness differences contributed less and changed little across scales. Environmental heterogeneity was positively associated with β‐diversity, primarily through replacement rather than richness differences. The variation explained by both environmental predictors and spatial structure increased with extent before approaching a plateau. Spatial structure explained the largest share of variation in BDtotal, Repl, and RichDif, while the relative importance of environmental predictors shifted with scale: soil variables were more influential at finer extents, whereas bioclimatic variables gained importance at broader extents. Environmental predictors were more strongly associated with species replacement than with richness differences, whereas spatial structure explained both components to a similar degree. Overall, increasing spatial extent amplified the magnitude of β‐diversity more than it altered the balance between its components, revealing species replacement as a scale‐robust feature of tropical forest metacommunities. These findings show that tropical forest β‐diversity emerges from the joint effects of environmental heterogeneity and spatial structure, and they highlight the need to conserve spatially dispersed, compositionally complementary habitat mosaics rather than focusing solely on local richness hotspots.

## Graphical abstract

Using a scale‐explicit metacommunity framework in a tropical rainforest, we examined how woody plant compositional dissimilarity and its replacement and richness‐difference components vary across spatial extents. Compositional dissimilarity increased modestly with extent but remained consistently dominated by species replacement, while environmental heterogeneity was more closely associated with replacement, and spatial structure imposed the strongest multiscale signal on both components. These findings provide a component‐resolved view of biodiversity scaling and clarify how environmental sorting and spatially structured processes jointly organize tropical forest metacommunities.

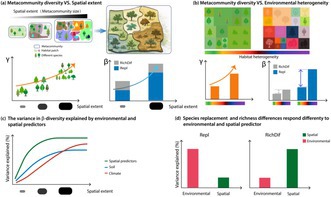

## 1 Introduction

Explaining how biodiversity is generated and maintained across space remains a central goal in ecology and biogeography (Whittaker et al. 1973; Levin 1992; Chave 2013; Bernardo‐Madrid et al. 2025). Local and regional diversity are linked by β‐diversity, which captures compositional differences among communities and thus provides a direct lens on the processes that structure assemblages (Whittaker 1960; Crist et al. 2003). A long‐standing challenge is to determine whether spatial variation in diversity is driven primarily by environmental heterogeneity, which expands niche opportunities, or by spatial processes such as dispersal limitation, historical contingency, ecological drift, biotic interactions, and species co‐occurrence structure (Tamme et al. 2010; Stein et al. 2014; Gianuca et al. 2017; Leibold and Chase 2018; Borthagaray et al. 2023). Because the relative importance of these mechanisms should vary with scale, understanding biodiversity patterns requires an explicitly scale‐dependent framework, especially in tropical forests where species richness is greatest and conservation stakes are highest (Bongalov et al. 2019; van Breugel et al. 2019; Parra‐Sanchez et al. 2023).

Environmental heterogeneity is expected to promote coexistence by increasing the range of habitats, resources and microclimates available to species (Grime 1973; Tilman 1982; Huston 1994; Macarthur 2008; Chen et al. 2020). Variation in climate, soils and topography can strengthen niche differentiation, increase richness, and enhance turnover among sites (Levine and HilleRisLambers 2009; Stein et al. 2014; He et al. 2022; Thomsen et al. 2022). Yet suitable habitats do not guarantee occupancy. Species must also disperse to those habitats and persist there, so diversity patterns can be shaped as much by spatial structure, species co‐occurrence patterns and assembly history as by measured environmental gradients (Leibold et al. 2004, 2021; Mateo et al. 2022). Metacommunity theory formalizes this view by treating local communities as components of a regionally connected network jointly governed by environmental sorting, dispersal, species interactions and ecological drift (Leibold et al. 2004, 2017; Leibold and Chase 2018; Viana and Chase 2019; Thompson et al. 2020). From this perspective, scale is not a nuisance but a mechanism: local environmental filtering may dominate at fine extents, whereas dispersal limitation and broad‐scale spatial structure may become increasingly important as extent expands (D'Amen et al. 2017; Banares‐de‐Dios et al. 2020; Tardanico and Hovestadt 2023). Although previous studies disentangled environmental and spatial processes from local to regional scales in tropical forests (Arellano et al. 2015), less is known about how β‐diversity components respond within dense reserve‐scale plot networks when metacommunity extent is varied continuously (Meynard et al. 2013; Arellano et al. 2015; He et al. 2023). A complementary approach is therefore to combine a continuous nearest‐neighbor gradient of metacommunity extent with explicit partitioning of total β‐diversity into species replacement and richness difference components.

Progress also depends on separating different forms of β‐diversity. Total compositional variation can arise through species replacement, where identities change among sites, or through richness differences, where sites differ in the number of species they contain (Baselga 2010; Podani and Schmera 2011; Legendre 2014). This distinction is mechanistically informative because the two components may not share the same drivers. Species replacement is often associated with environmental sorting and niche partitioning, whereas richness differences may reflect variation in regional species pools, dispersal limitation, broad‐scale filtering or area‐related effects (Chase and Myers 2011; Legendre 2014; Bae et al. 2020). Partitioning β‐diversity can therefore sharpen inference about community assembly, yet this approach has rarely been embedded within a metacommunity framework that explicitly examines scale dependence.

Tropical rainforests are an ideal but still underused system for such tests. They contain the highest terrestrial biodiversity on earth, but the processes governing spatial turnover in tropical forest communities remain incompletely resolved, particularly across scales (Condit et al. 2002; Wright 2002; Antão et al. 2019; Cooper et al. 2024). Here we examine how woody γ‐diversity, total β‐diversity, and its replacement and richness difference components changes with spatial extent in the Bawangling Nature Reserve, a key area of Hainan Tropical Rainforest National Park, China. Using a dense grid of vegetation plots, we assembled nested metacommunities by progressively aggregating neighboring plots across a continuous extent gradient. For each metacommunity, we quantified β‐diversity, and its replacement and richness difference components, and related these metrics to environmental heterogeneity and to spatial structure. This design allows us to ask three questions: how do total β‐diversity and its two components scale with extent; how is environmental heterogeneity associated with these diversity dimensions across scales; and how does the relative explanatory power of environmental predictors and spatial structure change with extent?

**FIGURE 1:**
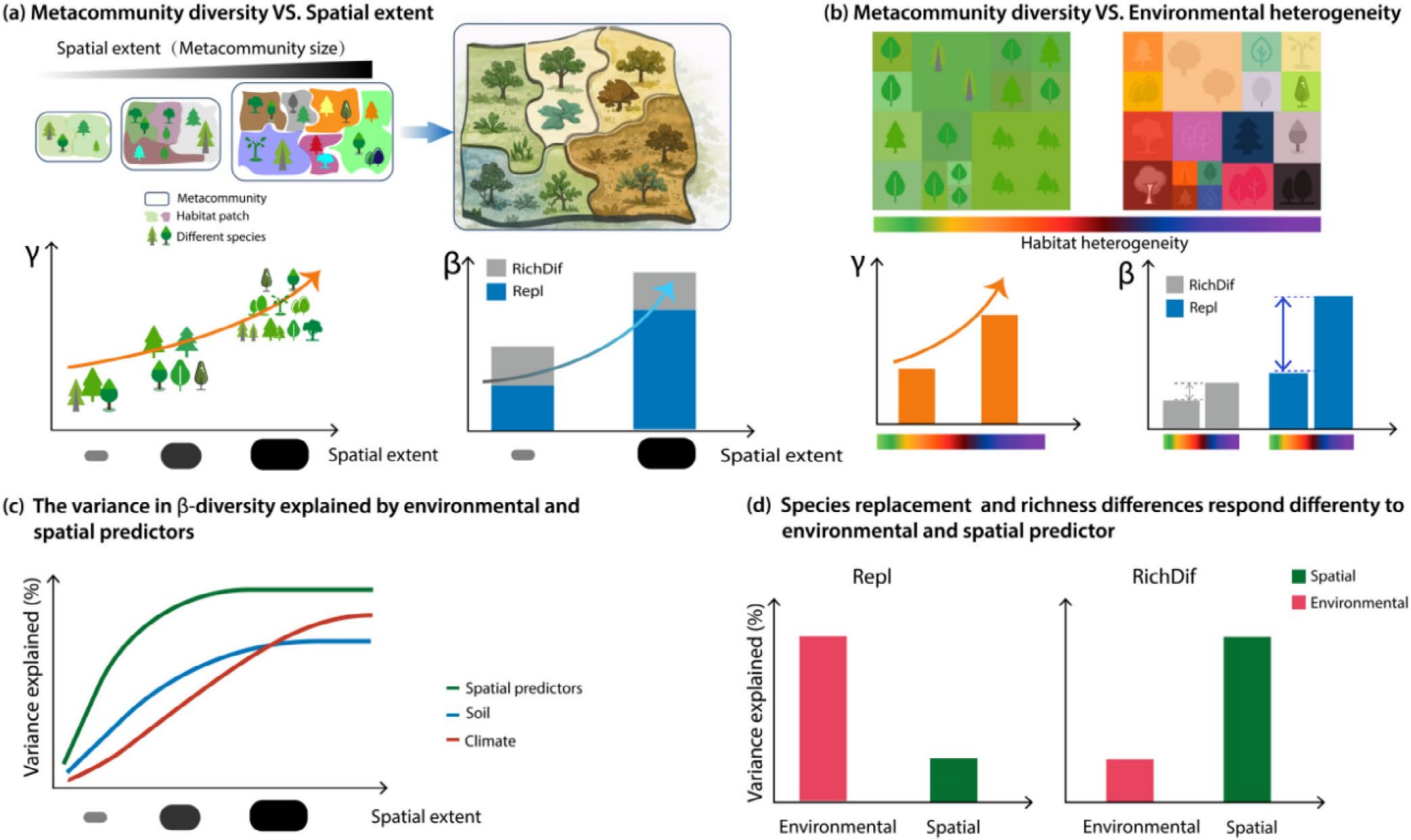
Conceptual framework illustrating scale‐dependent drivers of metacommunity β‐diversity and its components. This conceptual framework summarizes the hypothesized relationships among spatial extent, environmental heterogeneity, spatial structure, and metacommunity diversity across multiple scales. (a) As spatial extent increases, aggregating more local plots expands the regional species pool and incorporates compositionally distinct assemblages, leading to increases in γ‐diversity and β‐diversity, with compositional change expected to be dominated by species replacement rather than richness differences. (b) At a given spatial extent, greater environmental heterogeneity is predicted to increase both γ‐ and β‐diversity primarily through enhanced species replacement, reflecting species sorting along environmental gradients rather than strongly nested richness patterns. (c) With increasing spatial extent, the variance in β‐diversity explained by environmental and spatial predictors is expected to increase and then saturate, as a larger number of neighbors encompasses broader gradients but adds diminishing new information; spatial structure is expected to explain a substantial fraction of variation in total β‐diversity and its components due to dispersal limitation, historical processes, unmeasured environmental variation or spatially structured biotic processes. (d) Finally, species replacement and richness differences are hypothesized to respond differently to environmental and spatial predictors, with replacement more tightly linked to environmental gradients and richness differences more strongly associated with spatial structure and scale‐dependent constraints on habitat capacity.

We tested four hypotheses (Figure 1). First, metacommunity diversity should increase with spatial extent, and β‐diversity should be dominated by species replacement rather than richness differences, because larger extents incorporate more habitats and more compositionally distinct assemblages. Second, greater environmental heterogeneity should increase β‐diversity primarily through species replacement, as variation in climate and soils expands opportunities for niche differentiation. Third, the explanatory power of environmental predictors and spatial structure should increase with extent and then saturate, because larger metacommunities capture broader gradients but eventually add diminishing new information. Bawangling is characterized by complex terrain, strong environmental gradients and multiple forest types across a relatively compact landscape (Li et al. 2024, 2026), conditions under which dispersal limitation, historical assembly and unmeasured environmental variation may generate strong spatial structure. We therefore expect spatial structure to explain a substantial, and possibly larger, share of variation in β‐diversity than measured environmental variables; we also expect soil heterogeneity to be more important at finer extents and climatic heterogeneity to gain importance at broader extents. Fourth, the two β‐diversity components should differ in their dominant drivers: species replacement should track environmental gradients more closely, whereas richness differences should be more strongly associated with spatial structure, reflecting dispersal limitation and colonization‐extinction dynamics (Chase and Myers 2011; Legendre 2014; Bae et al. 2020).

## 2 Materials and Methods

### 2.1 Study Area

**FIGURE 2:**
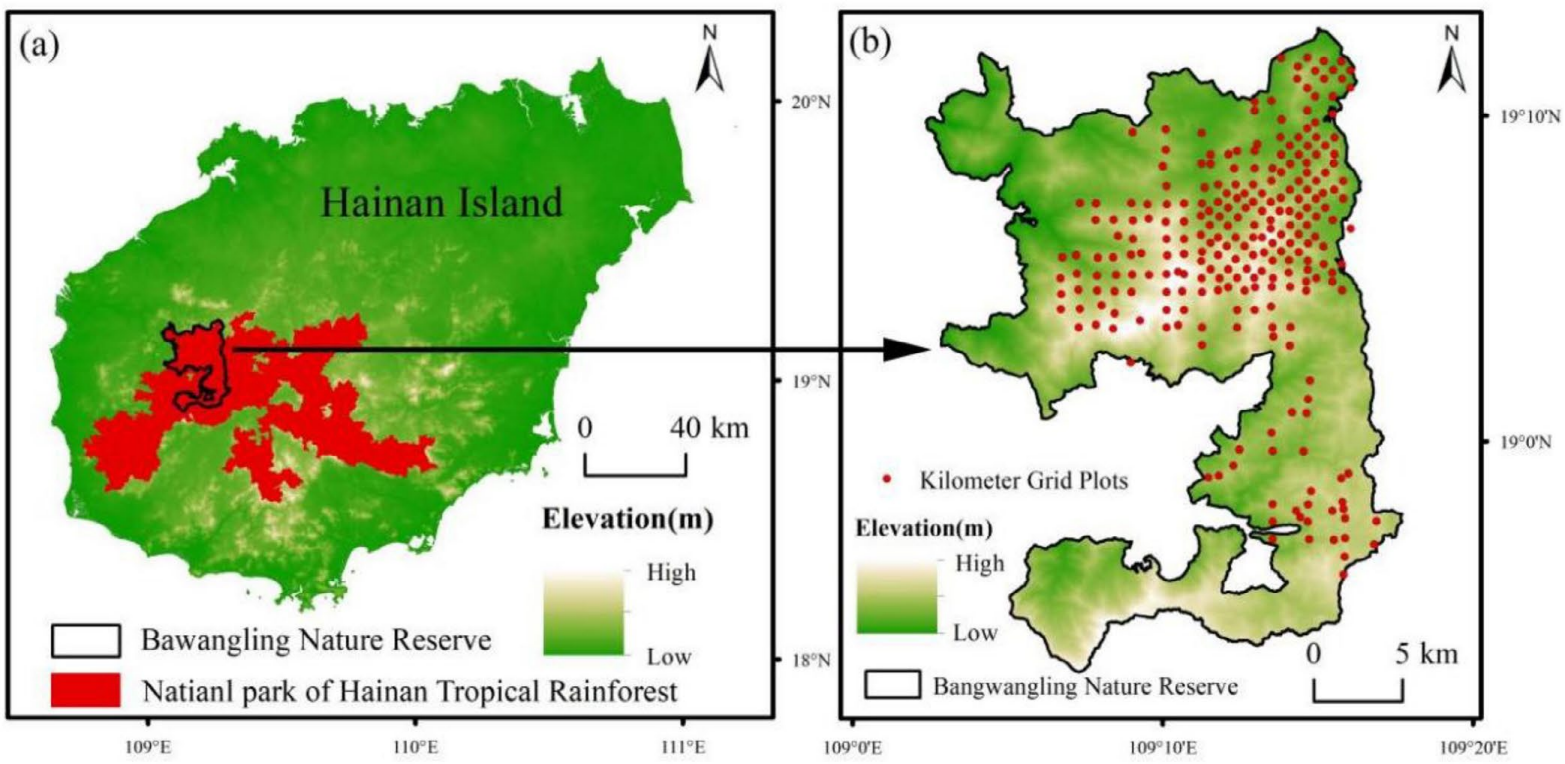
Location of the study area in the Bawangling Nature Reserve in China. (a) Location of Bawangling Nature Reserve of the National Park of Hainan Tropical Rainforest. (b) Distribution pattern of grid (1 × 1 km) sample plots in the Bawangling Nature Reserve. The black box outlines the boundary of the Bawangling Nature Reserve, while the red solid circles indicate the locations of the 20 m × 20 m sample plots within the nature reserve.

This study was carried out in Bawangling Nature Reserve, located within the National Park of Hainan Tropical Rainforest in Hainan Province, China (Figure 2), which is one of the first five national parks established in China. This area is characterized by a tropical monsoon climate, with elevations ranging from 123 to 1648 m. The mean annual precipitation is 2806 mm and the mean annual temperature is 23.6°C. The parent material for soil formation consists predominantly of a thick red weathering crust, and the soils are typified by the brick‐red soil type. The main vegetation types include tropical lowland rainforest, tropical monsoon rainforest, tropical coniferous forest, tropical montane rainforest, tropical montane evergreen forest and tropical hilltop dwarf forest (Li et al. 2026).

### 2.2 Experimental Design and Data Collection

#### 2.2.1 Vegetation and Environmental Data

A 1 × 1 km grid was placed in the study area and a 20 m × 20 m forest plot was randomly established within each cell (a total of 250 sample plots) following a standardized protocol (Figure 2). Plots were established and surveyed in 2020, and the community census methodology was identical for all sample plots: All woody stems with a diameter at breast height (DBH) of 1 cm or greater were measured for DBH, height and crown width and identified at the species level. The latitude and longitude coordinates of each plot were recorded with GPS in the center of the plot.

Previous studies in this study area have demonstrated the vital role of climate and soil properties in shaping plant diversity patterns (Li et al. 2024, 2026). Nineteen climate factors (bio1–bio19) were obtained from the website of WorldClim (http://www.worldclim.org/), with a spatial resolution of 30 arc sec. Nine soil variables were obtained from the Soil SubCenter, National Earth System Science Data Center, China National Science & Technology Infrastructure of China (http://soil.geodata.cn), with a horizontal spatial resolution of 90 m (Liu et al. 2022). The nine soil variables were pH, cation exchange capacity (CEC), bulk density (BD), coarse fragments (CF), total nitrogen (TN), total phosphorus (TP), total potassium (TK), soil thickness (ST), and soil organic carbon (SOC). Environmental values for each 20 m × 20 m plot were assigned by extracting the corresponding pixel values from the native‐resolution environmental rasters at the central coordinates of the plot.

#### 2.2.2 The Realization of Metacommunity at Different Spatial Extents

**FIGURE 3:**
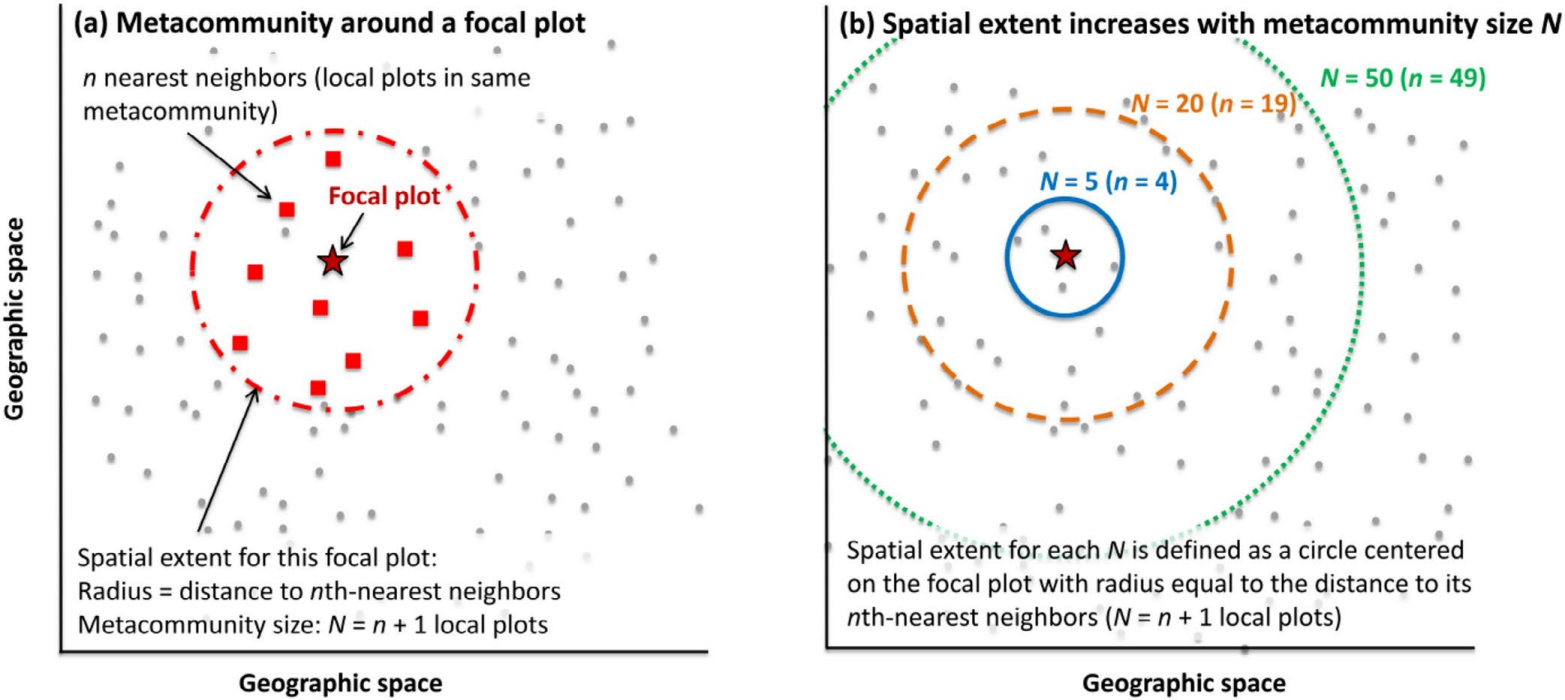
Conceptual illustration of metacommunity realization. (a) Local plots are treated as elements of a metacommunity. For a given focal plot (star), its *n* nearest‐neighbor plots (squares) are selected based on the geographic distance. Together, the focal plot and its *n* neighbors form a metacommunity with size *N* = *n* + 1 local communities. The spatial extent for this focal plot is operationally defined as a circle centered on the focal plot with radius equal to the distance to its *n*th‐nearest neighbor. (b) Example of how the spatial extent increases with metacommunity size *N* for the same focal plot (star). Concentric circles indicate the extents for *N* = 5, 20, and 50 (corresponding to *n* = 4, 19, and 49 nearest neighbors, respectively). Distances are computed from geographical coordinates, and the procedure is repeated for every plot and for *N*, with a range from 5 to 50.

Conceptually, we treated local plots as elements of a metacommunity sensu metacommunity theory (Leibold and Chase 2018). We constructed metacommunities by aggregating nearest‐neighbor plots (Xing and He 2019). For each iteration, one local plot was designated as the focal plot, and its *n* nearest plots were selected. The resulting metacommunity therefore contained *N* = *n* + 1 local communities—the focal community plus its *n* nearest neighbors (Zhang et al. 2020; Qiao et al. 2022) (Figure 3). We used *N* as an index of spatial extent; operationally, the extent for a given focal plot was the circle centered on that plot with a radius equal to the distance to its *n*th‐nearest neighbor. Distances were calculated from geographic coordinates and the procedure was repeated for every plot and for *N* ranging from 5 to 50 (i.e., *N* = 5–50 local plots). Diversity calculations and subsequent analyses were conducted at the metacommunity level.

#### 2.2.3 Environmental Heterogeneity Metrics and Spatial Structure

To evaluate environmental heterogeneity and diversity‐environmental heterogeneity relationships across spatial scales, we compiled two matrices of range values (maximum minus minimum) for environmental variables representing the properties of climate and soils. We quantified environmental heterogeneity at each spatial extent using two complementary metrics: the mean range of environmental variables (MeanRange) and the PCA − based heterogeneity metric (the standard deviation of PC1 scores, PC1_SD; for a detailed description of the methods, see Appendix S1). Across *N* = 5–50, PC1 explained 55.2%–63.1% of the variance in standardized environmental ranges (mean = 60.9%).

To model spatial structure, we computed distance‐based Moran's eigenvector maps (dbMEM) from the geographic coordinates of all local plots (Legendre and Legendre 2012). We retained only eigenvectors exhibiting positive spatial autocorrelation (Moran's > 0), and then used permutation‐based forward selection (*α* = 0.05 with the adjusted *R* ^2^ stop criterion) to avoid overfitting (Borcard and Legendre 2002; Dray et al. 2006; Legendre and Legendre 2012). The selected dbMEMs were therefore non‐correlated variables that can be used as spatial predictors in subsequent analyses. Ecologically, dbMEM eigenvectors do not represent individual measured environmental variables. Instead, they are spatial basis functions that describe broad‐ to fine‐scale spatial autocorrelation in the plot network. Variation explained by dbMEMs should therefore be interpreted as spatially structured community variation that may arise from dispersal limitation, historical assembly, spatially aggregated biotic interactions, or environmental gradients not included in the measured predictors (Legendre and Legendre 2012). We refer to this predictor group as spatial structure throughout the manuscript.

#### 2.2.4 Measures of Metacommunity Diversity

For each metacommunity realization and for each spatial extent, we define the metacommunity γ‐diversity as the inverse Simpson diversity of the pooled assemblage, that is, the Hill number of order *q* = 2: 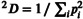, where *p*_*i*_ is the relative abundance of species *i* in the metacommunity formed by pooling *N* plots (Hill 1973; Chao et al. 2014). β‐diversity was quantified as the total variance (total sum of squares) of the site‐by‐species metacommunity matrices based on either presence‐absence or abundance data (BDtotal) (Legendre and De Caceres 2013; Legendre 2014). We partitioned β‐diversity, following the Podani‐family decomposition, into species replacement (Repl) and species richness difference components (RichDif) (Legendre 2014), using the beta.div.comp() function from the R package adespatial (Dray et al. 2012). Following the replacement–richness difference framework, we summarized the total β‐diversity and its additive components—species replacement and richness difference—for each realization of the metacommunity and for each *N*.

### 2.3 Data Analysis

#### 2.3.1 Scale‐Dependent Patterns of Metacommunity Diversity

To characterize how metacommunity diversity varies with spatial extent, we quantified its scale dependence along a continuous gradient in spatial extent. For each response variable (γ‐diversity, BDtotal, Repl, RichDif), we fitted an ordinary least squares model using all realizations across all spatial extents. To assess robustness to the choice of dissimilarity metric, we repeated the β‐diversity‐related analyses using (i) Sørensen dissimilarity for presence–absence data, (ii) Ružička dissimilarity for abundance data, and (iii) percentage difference dissimilarity for abundance data, partitioned following established abundance‐ and incidence‐based frameworks (Sorensen 1972; Baselga 2010, 2012; Legendre and De Caceres 2013; Legendre 2014). We applied a square‐root transformation to the species abundance data to stabilize the variance and reduce the influence of highly abundant species. These results (abundance‐based Jaccard, abundance‐based Sørensen, and presence–absence Sørensen and their decomposed components) exhibited qualitatively similar positive scale dependence; complete results are reported in Figures S2.1–S2.3. Therefore, for subsequent analyses, we rely exclusively on β‐diversity and its partitioned components calculated from Jaccard's dissimilarity coefficients based on the presence‐absence data.

#### 2.3.2 Diversity—Environmental Heterogeneity Relationships

We first quantified how environmental heterogeneity changes with spatial extent by regressing MeanRange and PC1_SD against metacommunity size. We then evaluated the relationships between diversity (γ‐diversity, β‐diversity and its components) and heterogeneity (MeanRange or PC1_SD) using ordinary least squares (OLS) regressions. Given the potential for spatial autocorrelation in our sampling design, we repeated our analyses and conducted additional diagnostics (Appendix S3). We first estimated spatial autoregressive (SAR) lag models (Kissling and Carl 2007). Residual maps of the SAR models indicated that the errors were not randomly distributed in space (Figure S3.1). Moreover, semivariograms of the SAR residuals suggested multiscale spatial structure not fully captured by models based solely on environmental heterogeneity (Figure S3.2), motivating spatial terms or spatially varying effects in subsequent analyses. To accommodate spatially varying effects suggested by the SAR diagnostics, we then fitted geographically weighted regression (GWR) models (LeSage 2004; Comber et al. 2022) to characterize the heterogeneity–diversity relationship (see Appendix S3 for details). The results of the GWR model indicate that the heterogeneity–diversity relationship was predominantly positive but spatially heterogeneous (Figures S3.3 and S3.4).

#### 2.3.3 Variation Partitioning and Scale‐Dependent Contributions of Environmental and Spatial Predictors

To quantify the relative contributions of environmental heterogeneity and spatial structure to β‐diversity and its decomposed components, we performed redundancy analyses (RDA) using three predictor groups: bioclimatic variables, soil variables, and spatial predictors derived from distance‐based Moran's eigenvector maps (dbMEM). For each extent of the metacommunity and each β‐diversity response (BDtotal, Repl, RichDif), we fitted separate RDAs for each predictor group and extracted adjusted *R* ^2^ as a measure of explained variation. This allowed us to track how the explanatory power of each predictor set varies with spatial extent. Prior to modeling, we mitigated collinearity using an iterative correlation filter (pairwise‐complete Pearson correlations): the variables were sequentially removed until the maximum |*r*| was < 0.7, each step dropping the variable most implicated in the strongest correlations. All retained predictors were standardized to mean 0 and unit variance (*z*‐scores). The abbreviations of the predictor variables and their corresponding descriptions are summarized in Table S1. To identify influential predictors within each group, we inspected the standardized coefficients of multivariate RDAs and univariate RDA models for individual variables; these results are summarized in Appendix S4.

We further compared the relative associations of predictors with species replacement and richness differences by computing Δ$R_{\mathrm{adj}}^{2}$ = $R_{\mathrm{adj}}^{2}$(Repl) − $R_{\mathrm{adj}}^{2}$(RichDif) for each predictor group. Positive Δ$R_{\mathrm{adj}}^{2}$ indicates a stronger association of replacement (Repl) with predictors, negative values indicate a stronger association of richness difference (RichDif), and values near zero imply comparable explanatory power. Plotting Δ$R_{\mathrm{adj}}^{2}$ across *N* allows us to assess the scale dependence and compare the differences between predictor sets.

All data preparation and statistical analysis was performed in R 4.4.1 (R Core Team 2024). All data and code used in this study are publicly available at the Zenodo repository (10.5281/zenodo.17763982).

## 3 Results

### 3.1 Scale‐Dependent Patterns of Metacommunity Diversity

**FIGURE 4:**
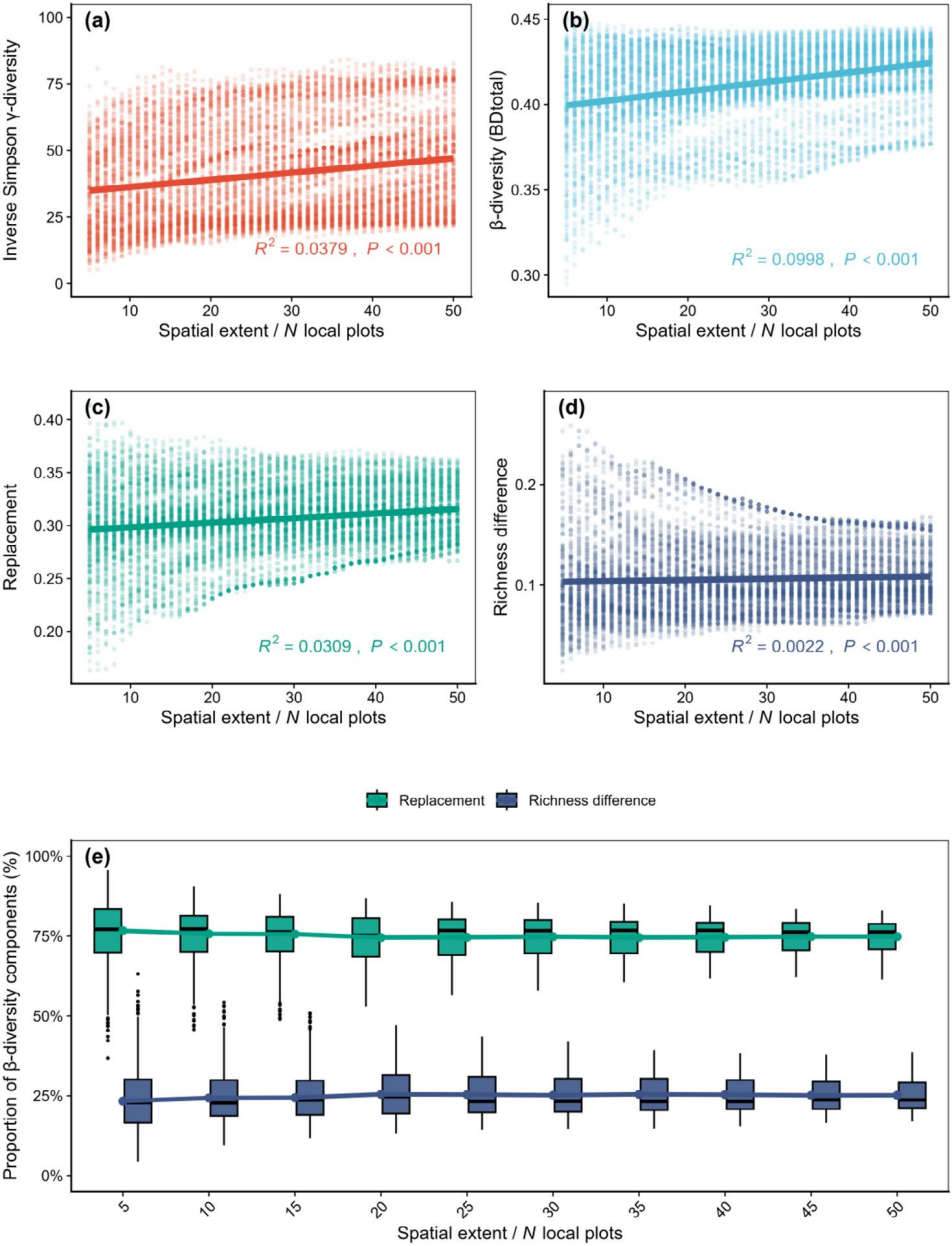
Scale dependence of metacommunity diversity. (a) Simpson γ‐diversity of the metacommunity as a function of spatial extent, expressed as the number of aggregated local plots (*N* = 5–50); (b) Total β‐diversity (BDtotal); (c) species replacement (Repl); (d) richness difference (RichDif) component of β‐diversity as functions of spatial extent. In panels (a–d), the points show all metacommunity realizations across spatial extents, and solid lines are ordinary least‐squares fits. The corresponding *R* ^2^ and *p* values are shown within each panel. (e) Proportional contributions of species replacement and richness difference to the total β‐diversity across spatial extents, shown as boxplots of the percentage of BDtotal explained by each component, with overlaid lines and points connecting the midpoints of the interquartile ranges.

Both γ‐ and β‐diversity increased with spatial extent (Figure 4; Figure S2.1—Figure S2.3). The results indicate that, along the spatial extent gradient, the inverse Simpson γ‐diversity exhibits a significant but modest positive relationship with spatial extents (*R* ^2^ = 0.038, *p* < 0.001; Figure 4a). BDtotal also shows a modest positive scale dependence (*R* ^2^ = 0.100, *p* < 0.001; Figure 4b). The decomposition of β‐diversity revealed a stronger contribution from species replacement (Repl) than from richness differences (RichDif), with Repl increasing with scale (*R* ^2^ = 0.031, *p* < 0.001; Figure 4c) and RichDif showing a very weak increase (*R* ^2^ = 0.0022, *p* < 0.001; Figure 4d). Consistently, across all spatial extents, the proportional contribution of replacement to β‐diversity remained high and remarkably stable (median 70 to 80% with relatively narrow interquartile ranges), while the richness difference accounted for a smaller and similarly invariant fraction of BDtotal (Figure 4e).

### 3.2 Scale Dependence of Environmental Heterogeneity and Diversity–Heterogeneity Relationships

**FIGURE 5:**
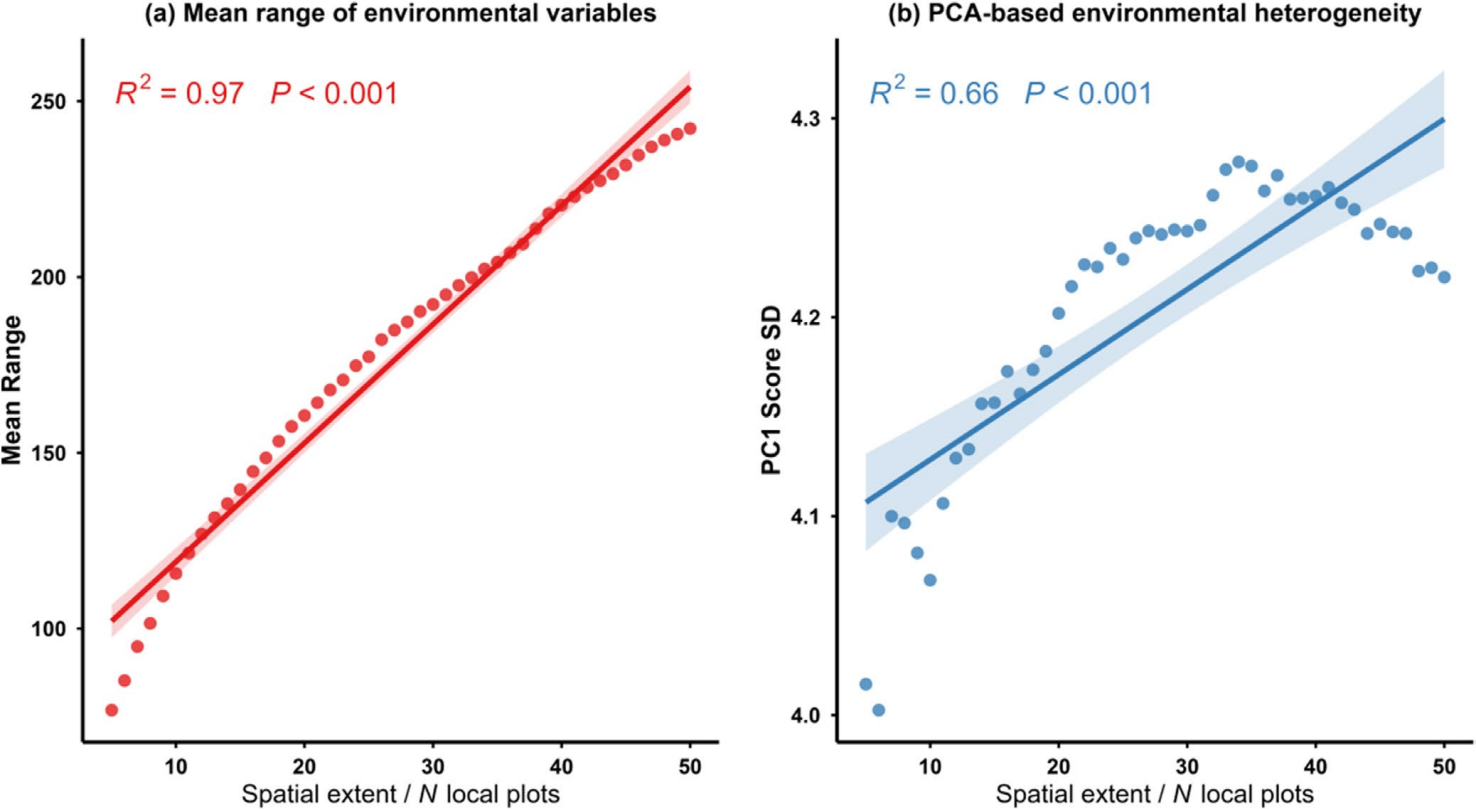
Environmental heterogeneity across spatial scales. (a) Mean range of environmental variables (MeanRange)—the scale‐level mean of per‐site range values (max—min) averaged across bioclimate and soil variables—plotted against spatial extent expressed as the number of local plots aggregated (*N* = 5–50). (b) PCA‐based heterogeneity (PC1 SD), defined as the standard deviation of PC1 scores from a PCA on standardized range matrices, versus *N*. Points denote scale‐specific estimates; solid lines show least‐squares fits with a 95% confidence interval.

Environmental heterogeneity increased overall with spatial extent (Figure 5). The mean range of environmental variables (MeanRange) increased almost linearly with the metacommunity size (*R* ^2^ = 0.970, *p* < 0.001) (Figure 5a). Multivariate heterogeneity (PC1_SD) also showed an overall positive relationship with spatial extent (*R* ^2^ = 0.660, *p* < 0.001) (Figure 5b), although the pattern was nonlinear: PC1_SD increased from small extents to approximately *N* = 35 and then flattened or slightly declined at the largest extents. Thus, aggregating more local plots generally broadened the climatic and edaphic gradients encompassed by each metacommunity, but the PCA‐based heterogeneity metric should not be interpreted as strictly linear across the entire extent gradient.

**FIGURE 6:**
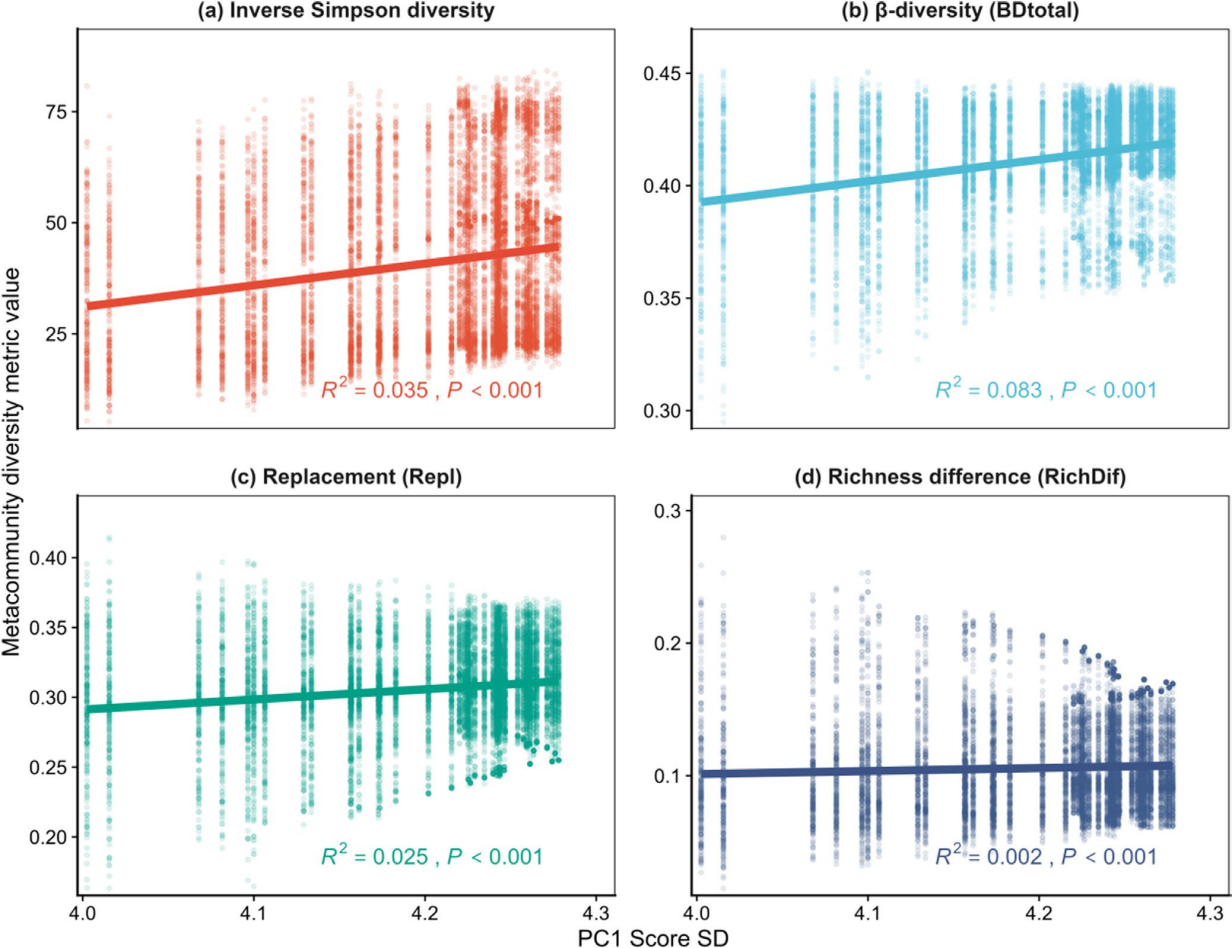
Relationships between environmental heterogeneity and diversity metrics. Panels show relationships between PC1_SD and (a) inverse Simpson γ‐diversity, (b) total β‐diversity (BDtotal), (c) species replacement (Repl), and (d) richness difference (RichDif). Points show metacommunity‐level observations; thick black lines show ordinary least‐squares fits. The annotations in each panel report *R* ^2^ and model significance (*p* < 0.001). PC1 score SD, the standard deviation of PC1 scores summarizing multivariate environmental heterogeneity based on bioclimatic and soil variables.

The inverse Simpson γ‐diversity (*R* ^2^ = 0.035, *p* < 0.001; Figure 6a) and total β‐diversity (*R* ^2^ = 0.083, *p* < 0.001; Figure 6b) both increased with environmental heterogeneity. Variation in total β‐diversity along the environmental heterogeneity gradient was primarily driven by the replacement component (*R* ^2^ = 0.025, *p* < 0.001; Figure 6c), whereas richness differences component exhibited a much weaker association (*R* ^2^ = 0.002, *p* < 0.001; Figure 6d). Spatial autoregressive models yielded similar patterns after accounting for residual spatial autocorrelation, and geographically weighted regressions further suggested that the strength of diversity–heterogeneity relationships varies across the landscape (Appendix S3, Figure S3.1—S3.4).

### 3.3 Scale‐Dependent Contributions of Environmental and Spatial Predictors

**FIGURE 7:**
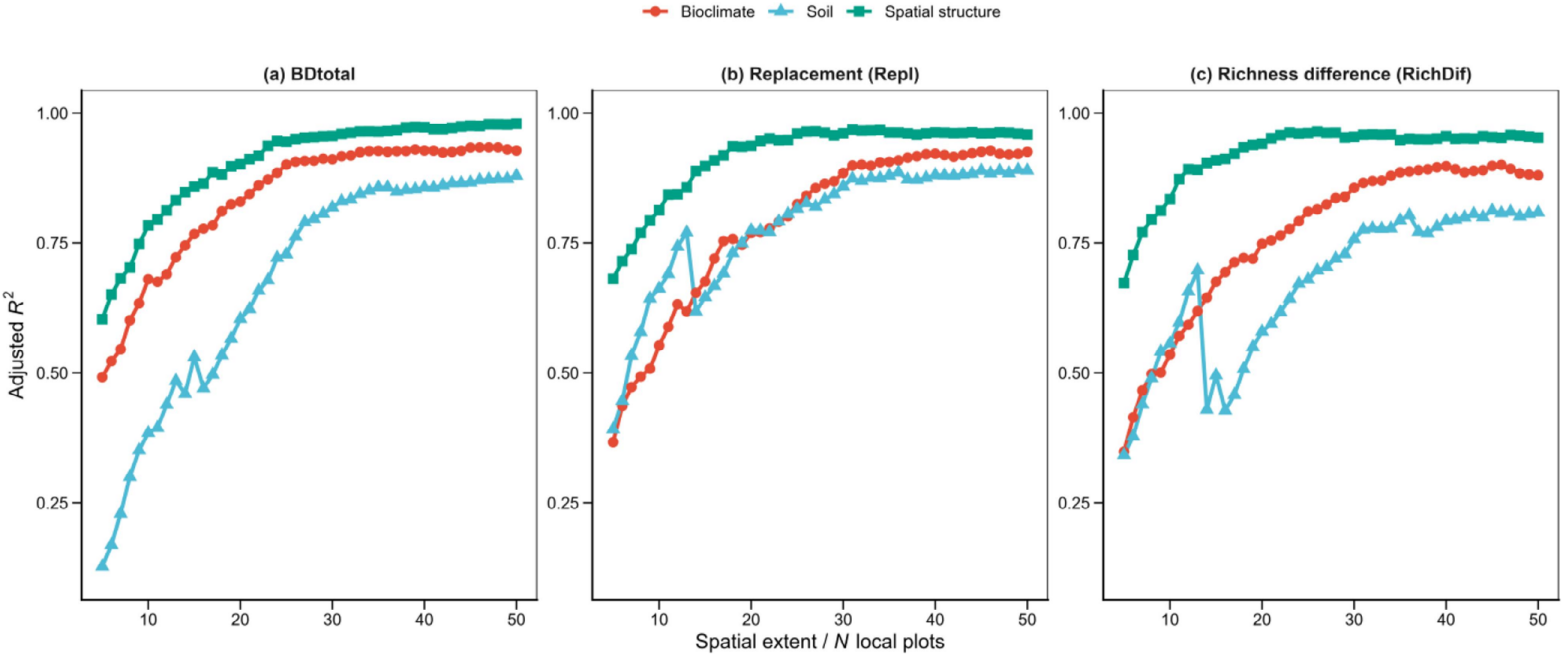
Adjusted *R* ^2^ of three‐group RDA models across spatial extents. Panels show response variables: (a) total β‐diversity (BDtotal), (b) species replacement (Repl), and (c) richness difference (RichDif). Lines and points show predictor sets for bioclimatic variables, soil variables, and spatial structure. Each environmental predictor set combines mean and range versions of variables after removal of highly collinear variables (|r| ≥ 0.7) and standardization. Spatial structure corresponds to dbMEM eigenvectors derived from plot coordinates.

Redundancy analyses revealed pronounced scale‐dependent gains in explained variation that rapidly approached asymptotes for all β‐diversity responses (Figure 7; Figure S4.1). The spatial structure (dbMEM) accounted for the largest share across the gradient, followed by the bioclimate and soil. The results showed a consistent saturation of explanatory power with increasing spatial extent and a dominant contribution of spatial structure, while bioclimate and soil provided substantial yet comparatively smaller effects. Soil explained more variation in Repl and RichDif than bioclimate did at small spatial extents; however, as spatial extent increased, the explanatory power of bioclimate rose and gradually surpassed that of soil for both components.

**FIGURE 8:**
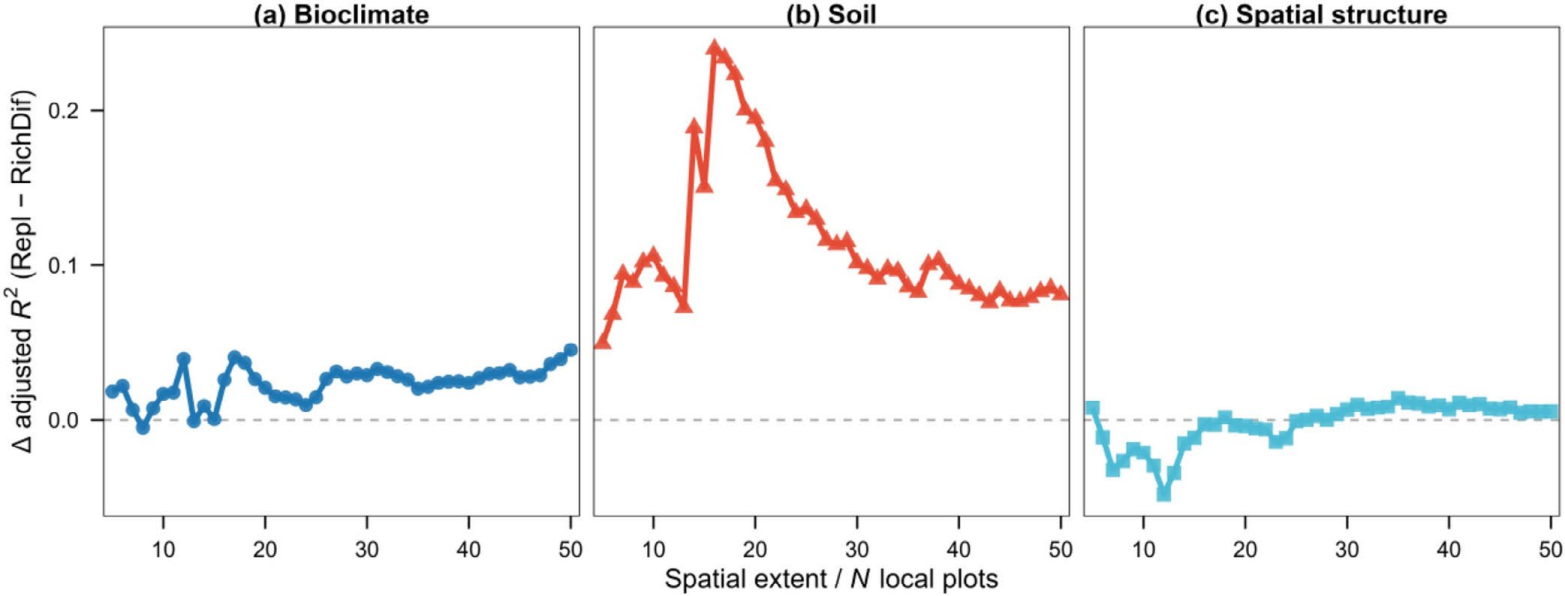
Difference in explanatory power between species replacement (Repl) and richness difference (RichDif). Δ$R_{\mathrm{adj}}^{2}$ = $R_{\mathrm{adj}}^{2}$(Repl) − $R_{\mathrm{adj}}^{2}$(RichDif); panels show (a) bioclimate variables, (b) soil variables, and (c) spatial structure. Each environmental predictor set combines mean and range versions of variables after removal of highly collinear variables (*|r*| ≥ 0.7) and standardization. Spatial structure corresponds to dbMEM eigenvectors derived from plot coordinates. The dashed line marks Δ$R_{\mathrm{adj}}^{2}$ = 0; positive values indicate greater explanatory power of replacement.

Within predictor groups, a limited subset of variables accounted for most of the explained variation (e.g., SOC and pH for soil, temperature‐ and moisture‐related bioclimatic variables, and leading dbMEM axes; Figures S4.2 and S4.3). We further compared the explanatory power of species replacement (Repl) versus richness difference (RichDif) across predictor groups and scales. The contrast between replacement and richness difference changes with spatial extent in a group‐specific, non‐linear manner (Figure 8). Environmental predictors, especially soil, were consistently more strongly associated with species replacement (Δ$R_{\mathrm{adj}}^{2}$ > 0 across spatial extents), while bioclimate showed a weaker but similar tendency. In contrast, Δ$R_{\mathrm{adj}}^{2}$ for spatial structure remained close to zero, indicating a broadly comparable explanatory power of the spatial structure for the replacement and richness differences.

## 4 Discussion

By combining a dense grid of woody‐plant plots with a nested metacommunity design, this study shows how the magnitude and drivers of β‐diversity change across a continuous gradient of spatial extent. In the Bawangling tropical rainforest, both γ‐ and β‐diversity increased with extent, but β‐diversity remained dominated by species replacement rather than richness differences. Environmental heterogeneity was positively related to diversity, but the relationships were modest and spatially non‐stationary. Spatial structure explained more variance than measured environmental predictors across most extents, although soils were relatively more important at fine extents and climate became more important at broader extents. Together, these patterns support a scale‐explicit metacommunity view in which niche‐based sorting and spatial processes interact to generate species replacement in tropical forests.

### 4.1 Scale Dependence and Replacement‐Dominated β‐Diversity

Both γ‐diversity and β‐diversity increased with spatial extent, but the slopes were shallow (Figure 4). Within a fine‐grained, reserve‐scale landscape, expanding neighborhood size appears to enlarge the pooled species set more than it alters the relative arrangement of local assemblages. Similar weak diversity‐extent relationships have been reported in other systems and are often attributed to restricted extent ranges and to the use of abundance‐weighted Hill numbers, which emphasize common rather than rare species (Chave 2013; Chao et al. 2014; Chase et al. 2018; Antão et al. 2019). In other words, the modest scale dependence observed here does not imply weak ecological structuring; rather, it suggests that much of the compositional organization of this forest is already expressed at relatively short distances.

More importantly, partitioning β‐diversity showed that species replacement accounted for roughly three‐quarters of total β‐diversity across all extents, whereas richness differences remained consistently small (Figures 4 and 6). This invariance indicates that adding plots mainly introduces different species assemblages rather than generating strong directional gradients in local richness. Such replacement‐dominated structure is broadly consistent with results from forests and from terrestrial, freshwater and marine systems, where species replacement usually explains much more β‐diversity than nestedness or richness differences (Si et al. 2015; Bergamin et al. 2017; Soininen et al. 2017; Bevilacqua et al. 2020; Fontana et al. 2020; Jiang et al. 2023; Ding et al. 2024; Yao et al. 2024). Mechanistically, this pattern is expected when environmental sorting and dispersal limitation continuously reshuffle species identities among sites without producing strong, monotonic richness gradients (Foster et al. 2010; Chase and Myers 2011; Leibold et al. 2017; Helm et al. 2024). For conservation, it means that complementarity among sites is likely to matter more than the protection of a few local richness hotspots (Socolar et al. 2016; Bergamin et al. 2017).

The relatively low *R* ^2^ values for Repl and RichDif compared with BDtotal also deserve attention. BDtotal integrates all compositional differences among sites and therefore accumulates signal from both replacement and richness differences. Once β‐diversity is partitioned, each component captures a narrower process and necessarily contains less of the total variance (Legendre 2014). The weak but significant extent effects on the components therefore suggest that spatial extent changes the overall magnitude of compositional dissimilarity more clearly than it changes the internal balance between species replacement and richness difference. This interpretation is consistent with the stable proportional contribution of replacement across scales.

### 4.2 Environmental Heterogeneity Explains Species Replacement, but Not Uniformly Across Space

As expected, both univariate and multivariate measures of environmental heterogeneity increased with spatial extent (Figure 5), indicating that aggregating plots broadened the climatic and edaphic gradients encompassed by each metacommunity. This result agrees with theory and evidence showing that environmental heterogeneity often co‐scales with area and expands ecological opportunity by increasing the volume of niche space available to species (Stein et al. 2014; Stein 2016). Diversity‐heterogeneity relationships were generally positive and the clearest signal emerged for the replacement component of β‐diversity (Figure 6). Thus, broader environmental gradients appear to promote species replacement more than they generate marked richness contrasts among plots. However, the low *R* ^2^ values, especially for Repl and RichDif, caution against interpreting these associations as strong deterministic evidence that measured heterogeneity alone defines viable niche space. Rather, the results indicate a weak but consistent statistical tendency that is likely mediated by additional unmeasured environmental, spatial and historical factors. This interpretation is consistent with the view that heterogeneity can increase the number of viable niches and thereby redistributes species identities across the landscape, but that its realized effect depends on dispersal, local context and the regional species pool (Pausas and Austin 2001; Thomsen et al. 2022; Jiang et al. 2023).

At the same time, the effects of heterogeneity were not spatially uniform. Residual spatial structure and geographically varying coefficients indicate that similar amounts of environmental variation can translate into different diversity responses in different parts of the reserve (Figures S3.1–S3.4). This spatial non‐stationarity likely reflects local context, including disturbance history, dispersal barriers, unmeasured environmental axes, species interactions, or variation in the surrounding species pool (Catano et al. 2017; van Breugel et al. 2019). Environmental heterogeneity is therefore important, but not sufficient, to explain diversity patterns in a complex tropical landscape. Its effects must be interpreted together with spatial structure and historical contingency (Dufour et al. 2006; Stein et al. 2014; Guo et al. 2017; Sola and Griffin 2025).

### 4.3 Relative Roles of Environmental Heterogeneity and Spatial Structure Across Scales

Redundancy analyses showed that explained variance rose rapidly with extent and then approached asymptotes for all β‐diversity responses (Figure 7; Appendix S4). Across most extents, spatial structure represented by dbMEM predictors explained more variation in total β‐diversity, replacement and richness differences than did measured bioclimatic or soil variables. This result is consistent with many variation‐partitioning studies in which spatial fractions equal or exceed environmental fractions in both forested and non‐forested systems (Murphy et al. 2015; Qiao et al. 2015; Gianuca et al. 2017; Oliveira et al. 2023). However, the strong spatial signal should not be interpreted as evidence that neutral or purely spatial processes alone dominate assembly. The so‐called pure spatial fraction inevitably absorbs several mechanisms at once, including dispersal limitation, historical colonization pathways, spatially structured species interactions and unmeasured but spatially structured environmental variation (Smith and Lundholm 2010; Gianuca et al. 2017; Yang et al. 2020; Lu 2021; He et al. 2023). In Bawangling, spatial structure therefore points to incomplete environmental characterization as well as genuine spatial structured constraints on community assembly, not to a rejection of niche‐based processes.

The environmental predictors themselves also changed in importance with scale. Soil variables explained more variation than climate at fine extents, whereas climatic predictors increased in importance and eventually exceeded soils at broader extents (Figure 7). This transition is ecologically plausible. Edaphic heterogeneity often operates over short distances and strongly structures composition among nearby plots, whereas broader neighborhoods are more likely to encompass gradients in temperature and moisture that elevate the role of climate (Stein et al. 2014; van Breugel et al. 2019; Jiang et al. 2023). The fact that a limited subset of variables captured most of the explained variance further suggests that β‐diversity in this forest is organized by a small number of dominant gradients embedded within a high‐dimensional spatial template. Additionally, the short‐lived change in soil adjusted *R* ^2^ around *N* = 15 coincided with changes in the collinearity‐filtered predictor sets: for example, SOC was dropped at *N* = 14, BD range entered at *N* = 15, and the number of retained soil predictors decreased by *N* = 16. We therefore interpret this local fluctuation as a model‐selection and collinearity‐filtering effect rather than as evidence for a sharp ecological threshold.

### 4.4 Replacement and Richness Differences Are Only Partly Decoupled

The partitioned analyses partly supported the prediction that replacement and richness differences should respond differently to environment and space. Environmental predictors, especially soils, explained more variation in replacement than in richness differences (Figure 8a,b), indicating that measured environmental gradients mainly determine which species occur where rather than how many species occur locally. This is the expected signature of species sorting along environmental gradients and reinforces the inference that species replacement in this system is strongly linked to niche‐based differentiation (Chase and Myers 2011; Legendre 2014; Gianuca et al. 2017).

By contrast, spatial predictors showed broadly similar explanatory power for replacement and richness differences, with Δ*R* ^2^ values oscillating around zero across scales (Figure 8c). This outcome suggests that spatial processes and spatially structured but unmeasured environments jointly influence both components of β‐diversity (Dray et al. 2006, 2012; Legendre and Legendre 2012; Gianuca et al. 2017; He et al. 2023). Dispersal barriers, historical contingencies, and fine‐scale habitat variation that was not directly measured may simultaneously alter species identities and constrain local richness. Under these conditions, there is no strong reason to expect spatial predictors to favor only one β component. More broadly, the results indicate partial, not complete, decoupling between replacement and richness differences: environmental heterogeneity is more closely aligned with species replacement, whereas spatial structure exerts comparable influence on both components.

### 4.5 Implications, Limitations and Future Directions

Two implications follow from these patterns. First, conservation planning in Bawangling and similar tropical forest landscapes should prioritize sets of sites that are environmentally complementary and sufficiently dispersed to capture compositionally distinct assemblages. Where β‐diversity is replacement dominated, protecting only local richness hotspots will miss a substantial fraction of the regional species pool (Bergamin et al. 2017; Gibson et al. 2017). In practice, this means that reserve planning should evaluate complementarity among sites across soil, climate and geographic gradients. Within large protected areas, management should maintain connectivity among environmentally distinct patches and avoid degradation of peripheral or compositionally unique habitats. In landscapes where one large reserve is not feasible, networks of smaller protected areas distributed across environmental gradients may complement large reserves, provided that connectivity and dispersal routes are maintained.

Second, the nested metacommunity approach has broader relevance beyond tropical forests. By examining how explained variance and β‐diversity components change with the number of aggregated sites, future studies can identify the spatial extent at which inference begins to stabilize. In our data, the RDA curves tended to level off around *N* ≈ 30 or larger, suggesting that studies based on very few local communities may detect transient or unstable patterns when the actual metacommunity extends across a larger spatial domain. This does not imply a universal minimum sample size because stabilization points will depend on habitat patchiness, dispersal distances, environmental gradients and taxon‐specific traits. It does, however, provide a practical strategy for evaluating whether sampling extent is sufficient for metacommunity inference: rather than choosing a single arbitrary scale, researchers can examine whether diversity components and explanatory power approach a plateau across increasing spatial extents.

Several limitations of this study highlight avenues for future research. The environmental dataset was restricted mainly to climate and soils, and did not include topography, canopy structure, disturbance history or biotic interactions, all of which could contribute to the unexplained spatial signal. The analysis focused on woody plants within a single protected area and on extents generated by aggregating 5–50 plots within a 1 × 1 km grid, so broader generalization to other taxa, landscapes and grain‐extent combinations remains to be tested. Finally, the analyses are observational and correlative. β‐diversity partitioning and variation decomposition are powerful for generating mechanistic inference, but they cannot decisively separate alternative processes that produce similar spatial patterns. Future work should integrate finer‐resolution environmental data, additional taxa, larger extents and temporal or model‐based approaches, including mechanistic metacommunity models and joint species distribution models, to test more directly the roles of filtering, dispersal limitation and stochasticity in tropical forests (D'Amen et al. 2017; Pichler and Hartig 2021; Guzman et al. 2022; Jackson et al. 2025; Nenzen et al. 2025). These future approaches are most valuable when used to compare mechanisms across spatial extents, rather than as methodological additions alone, because the central challenge is to determine whether inferred assembly processes remain stable as the spatial domain of the metacommunity changes.

## 5 Conclusions

Using a nested metacommunity framework built from an intensive grid of woody‐plant plots, we show that diversity in this tropical rainforest is weakly but consistently scale dependent. Both γ‐diversity and total β‐diversity increased with extent, yet β‐diversity remained overwhelmingly dominated by species replacement. Environmental heterogeneity in soils and climate was positively associated with diversity, especially with the replacement component, whereas spatial structure explained a large share of variation and often exceeded the measured environmental fractions. Soil heterogeneity was relatively more important at fine extents, whereas climate gained importance at broader extents. Together, these results indicate that tropical‐forest β‐diversity is produced by the interaction of environmental filtering, dispersal‐related spatial structure, and unmeasured spatially structured processes. More generally, the study shows that nested metacommunity designs and β‐diversity partitioning can reveal scale‐dependent mechanisms that would be obscured if diversity were analyzed only at a single extent or without separating species replacement from richness differences.

## Author Contributions

**Jie Yao:** conceptualization (equal), data curation (equal), funding acquisition (equal), methodology (equal), writing – original draft (equal), writing – review and editing (equal). **Xinran Li:** data curation (equal), investigation (equal), methodology (equal), writing – original draft (equal). **Shinichi Tatsumi:** conceptualization (equal), methodology (equal), writing – review and editing (equal). **Yi Ding:** data curation (equal), methodology (equal), writing – original draft (equal). **Jihong Huang:** writing – original draft (equal), writing – review and editing (equal). **Yue Xu:** writing – original draft (equal), writing – review and editing (equal). **Zhidong Zhang:** data curation (equal). **Wenxing Long:** data curation (equal). **Runguo Zang:** conceptualization (equal), funding acquisition (equal), project administration (equal), supervision (equal), writing – review and editing (equal).

## Funding

This work was supported by the National Key Research and Development Program of China (2023YFE0112801), Fund of CAS Key Laboratory of Forest Ecology and Silviculture, Institute of Applied Ecology, Chinese Academy of Sciences (KLFES‐2043), Fundamental Research Funds for the Central Non‐Profit Research Institution of CAF (CAFYBB2025MA022), National Natural Science Foundation of China (32101288).

## Conflicts of Interest

The authors declare no conflicts of interest.

## Supporting information


**Appendix S1:** The calculation of the environmental heterogeneity metrics (MeanRange and PC1_SD).
**Appendix S2:** Results of scale dependence of the β‐diversity and its decompose components for (i) Sørensen dissimilarity based on presence–absence data, (ii) Ružička dissimilarity based on abundance data, and (iii) percentage difference dissimilarity based on abundance data (including their decompose components).
**Appendix S3:** Results of spatial autoregressive modeling validation of diversity–heterogeneity relationships.
**Appendix S4:** Results of variation partitioning and scale‐dependent contributions of environmental and spatial predictors.
**Figure S2:1** Scale dependence of the β‐diversity and its decompose components using Sørensen dissimilarity based on presence–absence data.
**Figure S2:2** Scale dependence of the β‐diversity and its decompose components using Ružička dissimilarity based on abundance data.
**Figure S2:3** Scale dependence of the β‐diversity and its decompose components using percentage difference dissimilarity based on abundance data.
**Figure S3:1** Spatial distribution of residuals from the spatial autoregressive (SAR) model.
**Figure S3:2** Empirical semivariogram of model residuals.
**Figure S3:3** Geographically weighted regression (GWR) results for relationships between γ‐diversity and environmental heterogeneity.
**Figure S3:4** Geographically weighted regression (GWR) results for relationships between β‐diversity (BDtotal) and environmental heterogeneity.
**Figure S4:1** Adjusted *R* ^2^ of six single‐group RDA models across metacommunity size *N*, split by mean and range components within each predictor family.
**Figure S4:2** Heatmap of standardized coefficients from three‐group RDA models.
**Figure S4:3** Top 10 univariate predictors ranked by mean adjusted *R* ^2^.
**Table S1:** Predictor variables used in RDA analyses.

## Data Availability

All Data and R code supporting the results of this study are archived in Dryad (reviewer sharing link: https://doi.org/10.5061/dryad.1c59zw4bz).
