# Supplementary material for: Scale Dependence of Woody Plant β‐Diversity in a Tropical Rainforest Metacommunity
Source: Ecol Evol. 2026 Jul 12;16(7):e74022. doi: 10.1002/ece3.74022 (PMC13357691; doi:10.1002/ece3.74022)
Supplement: Supplementary file 1 — Appendix S1: The calculation of the environmental heterogeneity metrics (MeanRange and PC1_SD). Appendix S2: Results of scale dependence of the β‐diversity and its decompose components for (i) Sørensen dissimilarity based on presence–absence data, (ii) Ružička dissimilarity based on abundance data, and (iii) percentage difference dissimilarity based on abundance data (including their decompose components). Appendix S3: Results of spatial autoregressive modeling validation of diversity–heterogeneity relationships. Appendix S4: Results of variation partitioning and scale‐dependent contributions of environmental and spatial predictors. Figure S2:1 Scale dependence of the β‐diversity and its decompose components using Sørensen dissimilarity based on presence–absence data. Figure S2:2 Scale dependence of the β‐diversity and its decompose components using Ružička dissimilarity based on abundance data. Figure S2:3 Scale dependence of the β‐diversity and its decompose components using percentage difference dissimilarity based on abundance data. Figure S3:1 Spatial distribution of residuals from the spatial autoregressive (SAR) model. Figure S3:2 Empirical semivariogram of model residuals. Figure S3:3 Geographically weighted regression (GWR) results for relationships between γ‐diversity and environmental heterogeneity. Figure S3:4 Geographically weighted regression (GWR) results for relationships between β‐diversity (BDtotal) and environmental heterogeneity. Figure S4:1 Adjusted R 2 of six single‐group RDA models across metacommunity size N, split by mean and range components within each predictor family. Figure S4:2 Heatmap of standardized coefficients from three‐group RDA models. Figure S4:3 Top 10 univariate predictors ranked by mean adjusted R 2. Table S1: Predictor variables used in RDA analyses. [file ECE3-16-e74022-s001.docx]

Title: Scale dependence of woody plant β-diversity in a tropical rainforest metacommunity

**Supporting Information**

# **The calculation of the environmental heterogeneity metrics**

To assess how the environmental heterogeneity and the diversity–environmental heterogeneity relationships varied across spatial scales, we established two complementary metrics to evaluate environmental heterogeneity based on environmental variables (including bioclimatic and edaphic factors). For both methods, we first calculated the range (i.e., maximum minus minimum) of each environmental variable across the neighborhoods defined by different spatial scales (N = 5 to 50). These range values were used to quantify multivariate environmental variability.

1. *****Mean range of environmental variables*****

We calculated the mean of all environmental ranges at each spatial scale, resulting in a univariate heterogeneity index representing the average environmental divergence across neighboring plots. This approach captures the general magnitude of environmental variation without accounting for covariation among variables. For site *i* at scale *N*, we first averaged the variable-wise ranges:

${\bar{\text{R}}}_{\text{iN}}\text{=}\frac{\text{1}}{\text{p}}\text{ }\sum_{\text{v}\text{=1}}^{\text{p}} \text{R}_{\text{i}\text{vN}}$

where $\text{R}_{\text{i}\text{vN}}$ is the range of variable *v* for site *i* at scale *N*, and *p* is the total number of environmental variables. We then averaged across sites to obtain the scale-level metric:

$\text{MeanRange}_{\text{N}}\text{=}\frac{\text{1}}{\text{m}}\text{ }\sum_{\text{i}\text{=1}}^{\text{m}} {\bar{\text{R}}}_{\text{iN}}$

with *m* the number of sites at scale *N*.

1. *****PCA-based heterogeneity*****

We applied principal component analysis (PCA) to the matrix of environmental range variables at each neighborhood size separately. For each PCA, we extracted the scores along the first principal component (PC1), which represents the dominant axis of multivariate variation. We then calculated the standard deviation of PC1 scores among all plots at that scale. This value serves as a synthetic multivariate heterogeneity index that reflects both the magnitude and structure of environmental variation. Unlike the mean range, this PCA-based measure considers the correlation structure among variables and emphasizes the most informative gradient in the environmental space. At each scale *N*, we *z*-standardized variables and ran PCA. We extracted the first principal component scores $\text{s}_{\text{iN}}$ and defined

${\text{PC}\text{1\_}\text{SD}}_{\text{N}}\text{=sd(}\text{s}_{\text{iN}}\text{)}$

i.e., the across-site standard deviation of PC1 scores, which summarizes multivariate environmental spread.

# Results of scale dependence of the β-diversity and its decompose components for (i) Sørensen dissimilarity based on presence–absence data, (ii) Ružička dissimilarity based on abundance data, and (iii) percentage difference dissimilarity based on abundance data (including their decompose components).


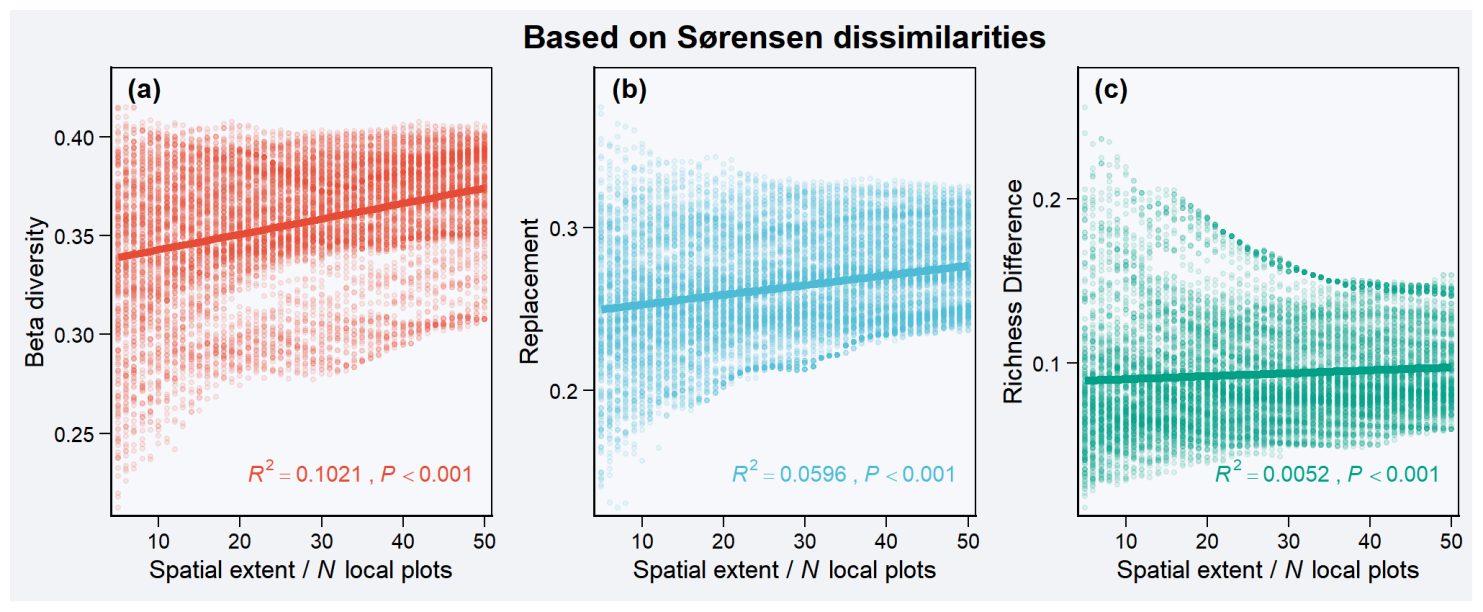


Figure S2.1 ****Scale dependence of the β-diversity and its decompose components.**** As in Fig. 3 in main test, but using Sørensen dissimilarity based on presence–absence data and its standard two-component partition. Points indicate all realizations; solid lines are ordinary least-squares (OLS) fits with 95% confidence intervals. Patterns mirror those in the main text, with replacement contributing more strongly than richness differences across scales.


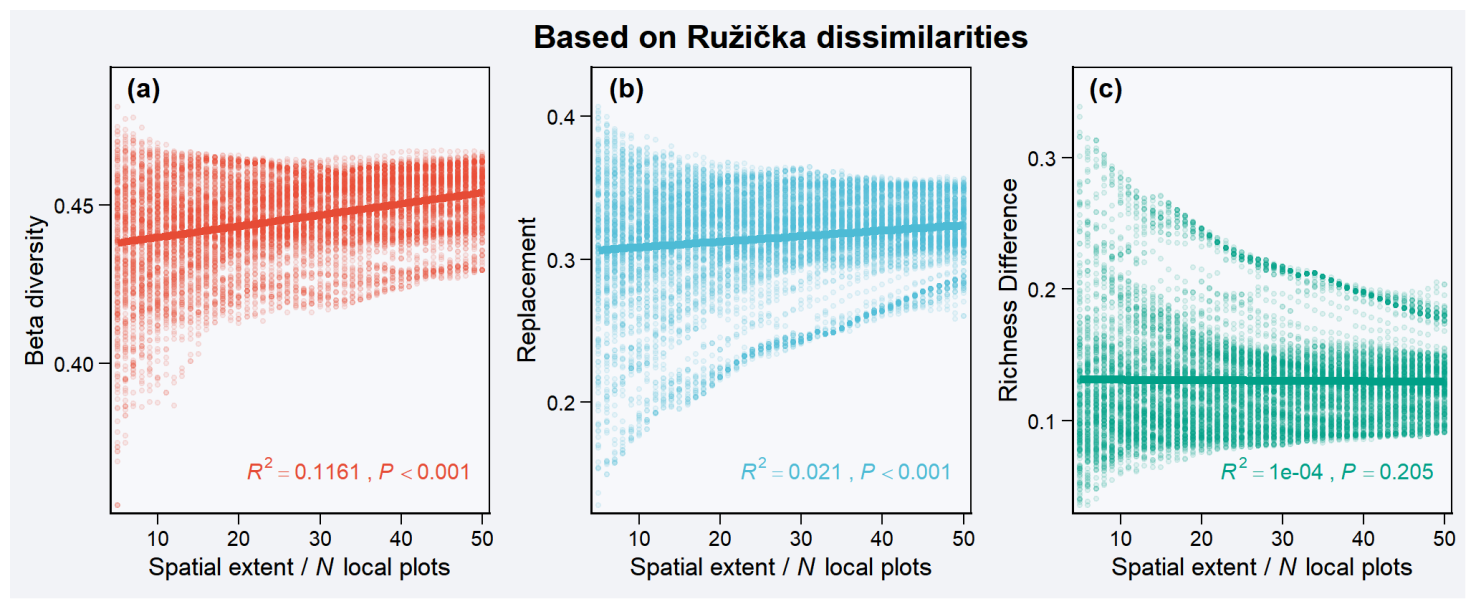


Figure S2.2 ****Scale dependence of the β-diversity and its decompose components.**** As in Fig. 3 in main test, but using Ružička dissimilarity based on abundance data, and its standard two-component partition. Points indicate all realizations; solid lines are ordinary least-squares (OLS) fits with 95% confidence intervals. Results corroborate the main-text pattern of shallow positive scaling with the spatial extent *N*.


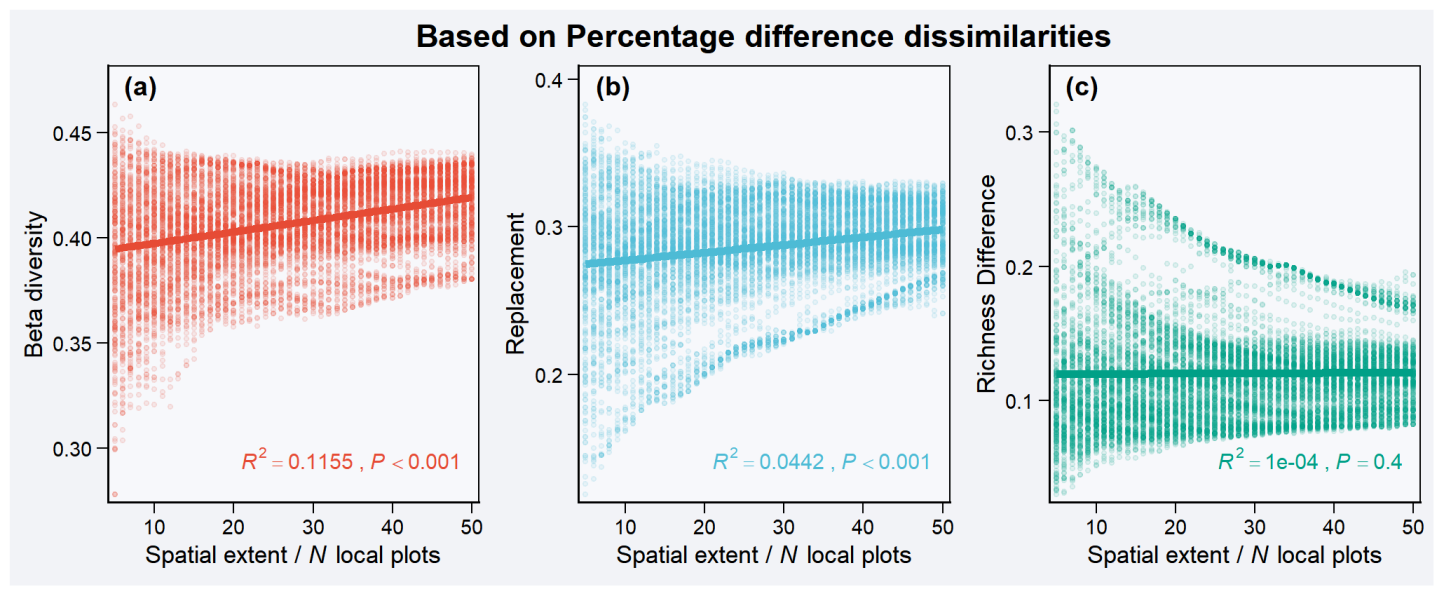


Figure S2.3 ****Scale dependence of the β-diversity and its decompose components.**** As in Fig. 3 in main test, but using percentage difference dissimilarity based on abundance data and its standard two-component partition. Points indicate all realizations; solid lines are ordinary least-squares (OLS) fits with 95% confidence intervals. Trends are weakly positive with *N*, broadly consistent with presence–absence results.

# Results of spatial autoregressive modeling validation of diversity–heterogeneity relationships

To account for potential spatial autocorrelation in the relationship between metacommunity diversity and environmental heterogeneity, we employed spatial autoregressive (SAR) lag models and geographically weighted regression (GWR) models. The response variables were inverse Simpson diversity (*γ*-diversity), β-diversity (*BDtotal)*, and two predictors were considered independently: the mean environmental range (MeanRange) and the standard deviation of the first principal component of environmental variables (PC1_SD).

SAR lag models were fitted using the lagsarlm() function from the **spatialreg** package in R (Bivand, Millo, & Piras, 2021). Spatial weights were constructed using a 5-nearest-neighbors (k = 5) approach based on the geographic coordinates (longitude and latitude) of each forest plot. To visualize model fits, we created bivariate scatterplots showing the relationships between *γ-diversity*, *BDtotal* and each predictor. To assess residual spatial structure, we computed empirical semivariograms of model residuals using geographic coordinates for each plot. We used the classical Matheron estimator, binning pairwise distances into equally spaced lags up to ~50% of the maximum inter-point distance (12 bins by default). To accommodate spatially varying effects suggested by the SAR diagnostics, we fitted geographically weighted regression (GWR) models for the environmental heterogeneity–diversity relationship using two alternative heterogeneity metrics (mean range across environmental variables and the SD of PC1 scores). We used an adaptive kernel with bandwidth selected by AICc via leave-one-out cross-validation. We mapped local coefficients and local R² and compared the two specifications by (i) the distribution of local R² and (ii) the proportion of plots where one specification achieved higher local R². Interpretation focused on spatial patterns in local effects and goodness-of-fit.

Using spatial autoregressive (SAR–lag) models, we evaluated the association between environmental heterogeneity and metacommunity diversity for both γ‐diversity and β‐diversity. The residual maps show that, after accounting for spatial lag dependence, model errors are generally small and spatially mottled across much of the region, but coherent patches of underestimation (red) and overestimation (blue) persist in localized areas (Fig. S3.1). This indicates that SAR substantially reduces, but does not completely remove, spatial structure in the response.


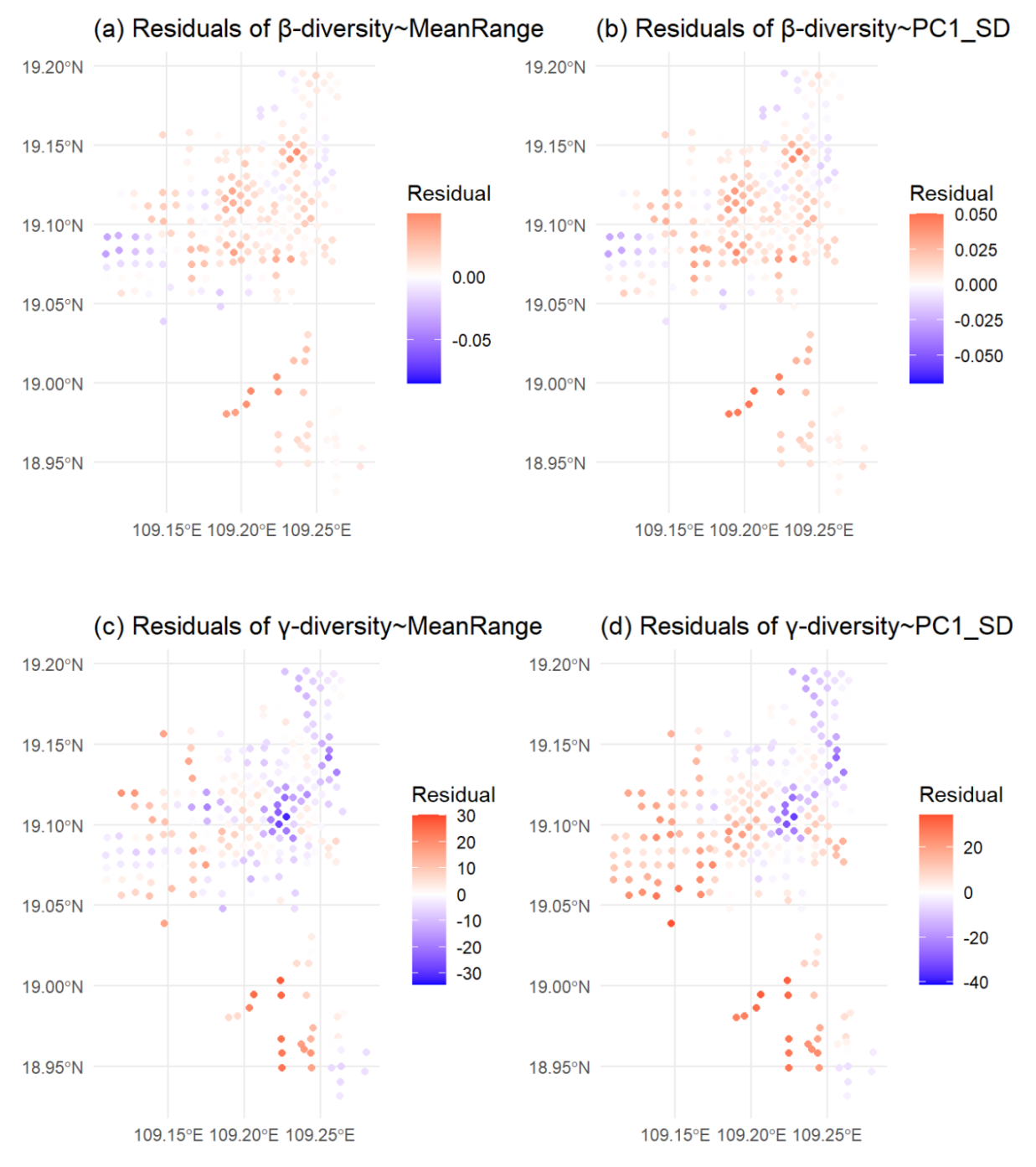


Figure S3.1 **Spatial distribution of residuals from the spatial autoregressive (SAR) model.** Points are plotted at plot centroids (Longitude, Latitude). Colors denote residual sign and magnitude (red = underestimation, blue = overestimation). Mean Range, the mean range of environmental variables, the range values (max − min) for environmental variables representing climate and soils properties; PC1_SD, the standard deviation of PC1 scores summarizing multivariate environmental heterogeneity.

The empirical semivariograms of SAR residuals further support this interpretation. For γ‐diversity, semivariance rises steeply at short lags and approaches a sill within relatively short distances, consistent with short‐range spatial autocorrelation that is largely attenuated by the SAR–lag specification (Fig. S3.2). In contrast, for β‐diversity the semivariogram shows a more gradual increase and a broader effective range, implying residual spatial structure at larger scales (Fig. S3.2). Together, these diagnostics suggest that while heterogeneity explains a meaningful component of spatial variation in diversity, unmodeled broad‐scale structure and/or spatial non-stationarity remain—particularly for β‐diversity—motivating the complementary GWR analyses.


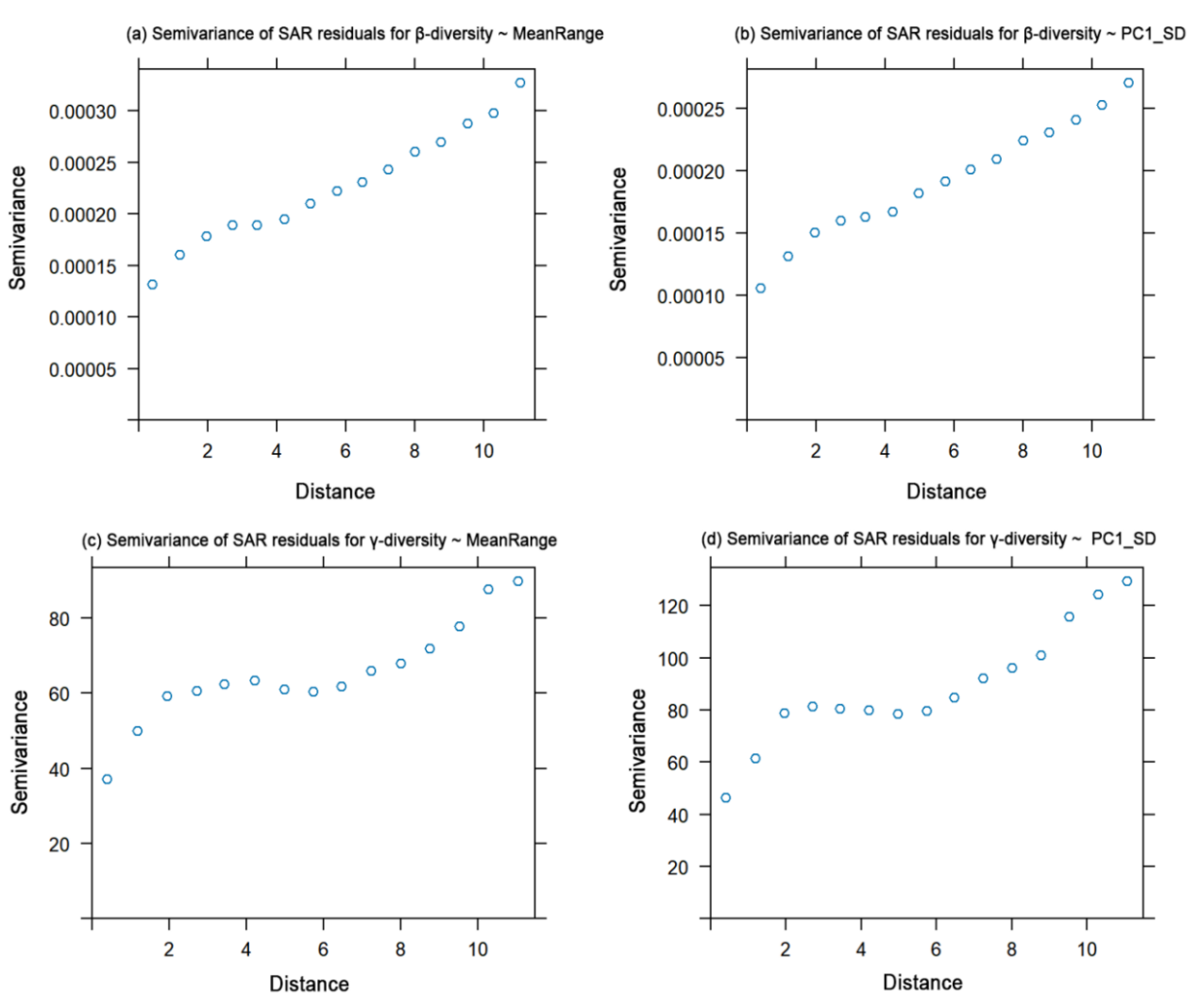


Figure S3.2 **Empirical semivariogram of model residuals**. Semivariance (points) is plotted against binned inter-plot distance. Mean Range, the mean range of environmental variables, the range values (max − min) for environmental variables representing climate and soils properties; PC1 _SD, the standard deviation of PC1 scores summarizing multivariate environmental heterogeneity.

Geographically weighted regression revealed clear spatial non-stationarity in the relationships between environmental heterogeneity and metacommunity diversity for both γ- (Fig. S3.3) and β-diversity (Fig. S3.4). Across plots, most local slopes were positive: for γ-diversity, 86% of locations showed positive coefficients for MeanRange and 85% for PC1_SD; for β-diversity the corresponding shares were 88% and 83%. Median local effect sizes were positive for all models (γ: MeanRange median = 0.076, PC1_SD median = 51.01; β: MeanRange median = 1.42×10⁻⁴, PC1_SD median = 0.084), but the interquartile ranges (IQRs) were wide and coefficients spanned negative to positive values (e.g., γ: PC1_SD range −146 to 182), indicating strong geographic variation in both the strength and, locally, the sign of the associations.

Model fit varied substantially across space. Median local *R*^2^ was moderate to high for all models—γ: 0.57–0.64; β: 0.56–0.69—with broad IQRs (γ: 0.31–0.46; β: 0.37–0.41) and maxima >0.90, highlighting hotspots where heterogeneity explained a large proportion of local diversity variation and pockets where the relationship was weak. Taken together, the GWR results indicate that the heterogeneity–diversity relationship is predominantly positive but spatially heterogeneous, with stronger and more consistent effects in some subregions and weaker or even negative effects elsewhere—patterns consistent with the residual spatial structure observed in the SAR diagnostics.


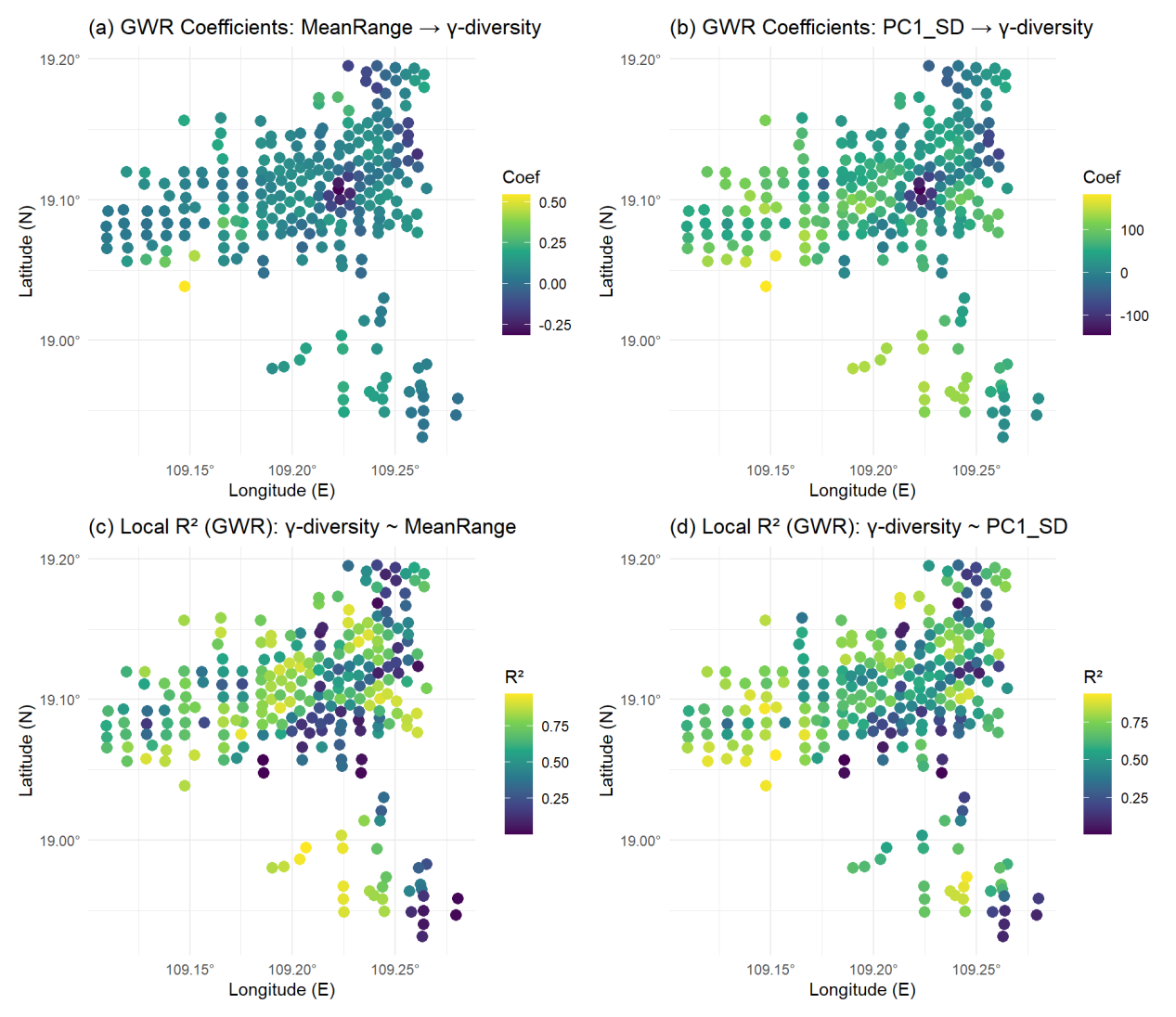


Figure S3.3 ****Geographically weighted regression (GWR) results for relationships between γ‐diversity and environmental heterogeneity.**** Maps show spatially varying relationships estimated by GWR: (a) local coefficients for **MeanRange and γ‐diversity**; (b) local coefficients for **PC1_SD and γ‐diversity**; (c–d) corresponding **local** *R*^2^. Points are plot centroids (Longitude, Latitude). Warm colors indicate stronger positive effects (cool colors negative); higher local *R*^2^ denotes greater local explanatory power. Note that coefficient magnitudes are not directly comparable between predictors because the two heterogeneity metrics are on different scales. Mean Range, the mean range of environmental variables, the range values (max − min) for environmental variables representing climate and soils properties; PC1_SD, the standard deviation of PC1 scores summarizing multivariate environmental heterogeneity.


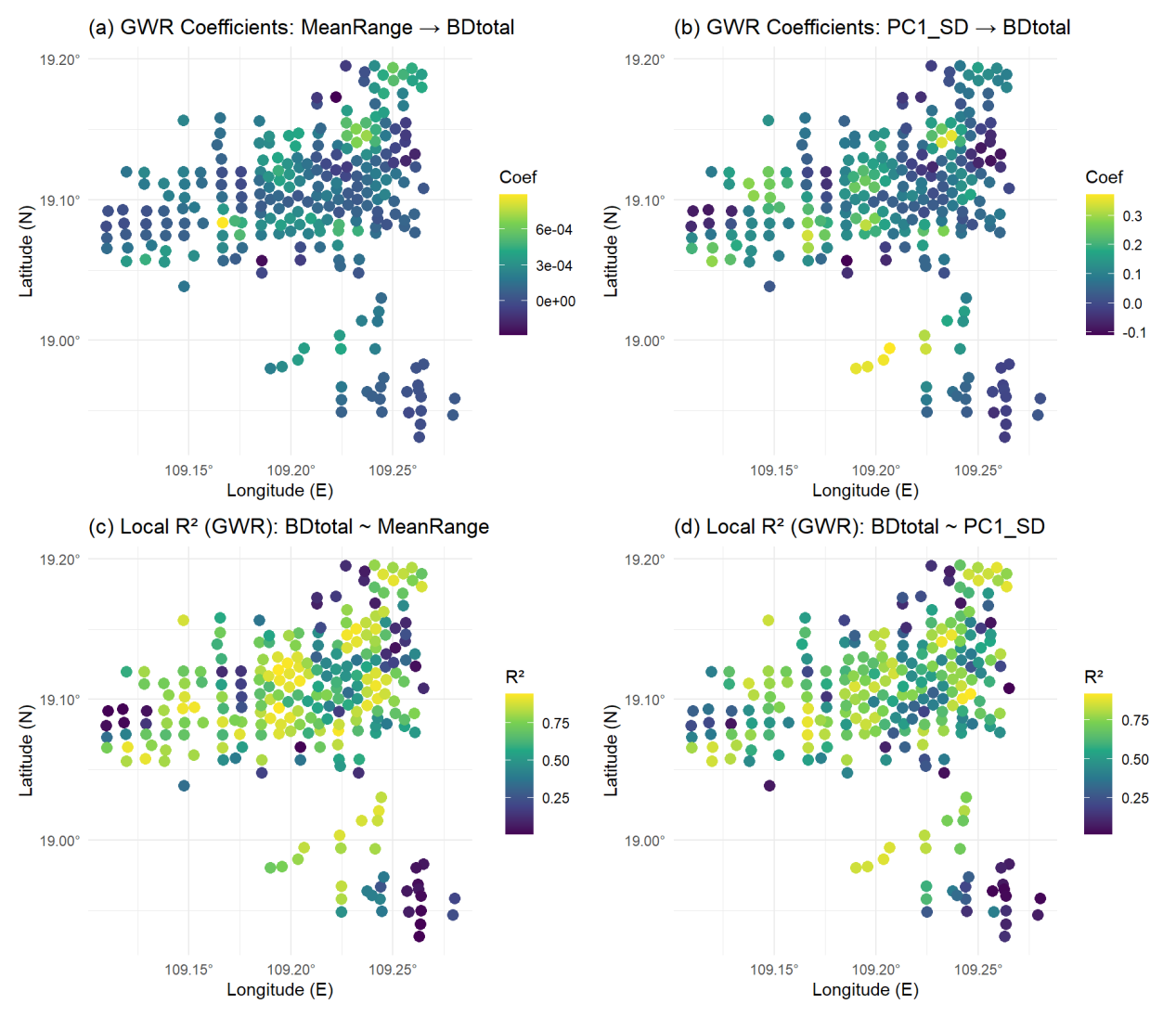


Figure S3.4 ****Geographically weighted regression (GWR) results for relationships between β‐diversity (BDtotal) and environmental heterogeneity.**** As in Figure S3.3, panels depict (a) local coefficients for **MeanRange and BDtotal**; (b) local coefficients for **PC1_SD and BDtotal**; (c–d) **local** *R*^2^ maps. Colors encode effect sign and magnitude (coefficients) or goodness of fit (local *R*^2^). Note that coefficient magnitudes are not directly comparable between predictors because the two heterogeneity metrics are on different scales. Mean Range, the mean range of environmental variables, the range values (max − min) for environmental variables representing climate and soils properties; PC1 Score SD, the standard deviation of PC1 scores summarizing multivariate environmental heterogeneity.

# Results of variation partitioning and scale-dependent contributions of environmental and spatial predictors

We partitioned the variance of total β-diversity (BDtotal) and its components—replacement (Repl) and richness difference (RichDif)—attributable to bioclimate, soil, and dbMEM. To disentangle the roles of **central tendency** vs **within‐metacommunity variability** in environmental and spatial predictors, we fitted six single–predictor–set models (bioclimate_mean, bioclimate_range, soil_mean, soil_range, dbMEM_mean, dbMEM_range) across spatial extents.

Adjusted *R*^2^ increased sharply at small spatial extents and approached asymptotes for all responses (BDtotal, Repl, RichDif), with **spatial structure** dominating the absolute explanatory power (Figure S4.1). Averaged across *N*, dbMEM_mean achieved the highest adjusted *R*^2^, followed by dbMEM_range and bioclimate_mean. Soil models were lower but still substantial (soil_mean: 0.557, 0.652, 0.541; soil_range: 0.511, 0.658, 0.435). Permutation tests (999 permutations) indicated significance for all models (*p* ≤ 0.002 across groups and responses). Collectively, these patterns reveal consistent saturation of explanatory power with increasing *N* and a **dominant contribution of spatial structure**, with bioclimate and soil providing substantial but comparatively smaller effects.

Contrasting **mean** vs **range** components within each predictor family clarified their relative contributions (Figure S4.1). For **bioclimate**, mean variables outperformed range variables for all responses (mean − range in adjusted *R*^2^: **+0.387** for BDtotal, **+0.096** for Repl, **+0.121** for RichDif). For **dbMEM**, mean also exceeded range (**+0.064**, **+0.083**, **+0.059**, respectively). For **soil**, the picture was mixed: mean > range for BDtotal (**+0.046**) and RichDif (**+0.106**), while range slightly exceeded mean for Repl (**−0.006** on average), in line with the visually higher Repl curve for soil_range at intermediate *N*. These comparisons indicate that central tendencies of climate and spatial gradients explain more variance than within–metacommunity variability per se, whereas **soil variability can be comparably or more related to turnover** at some scales.


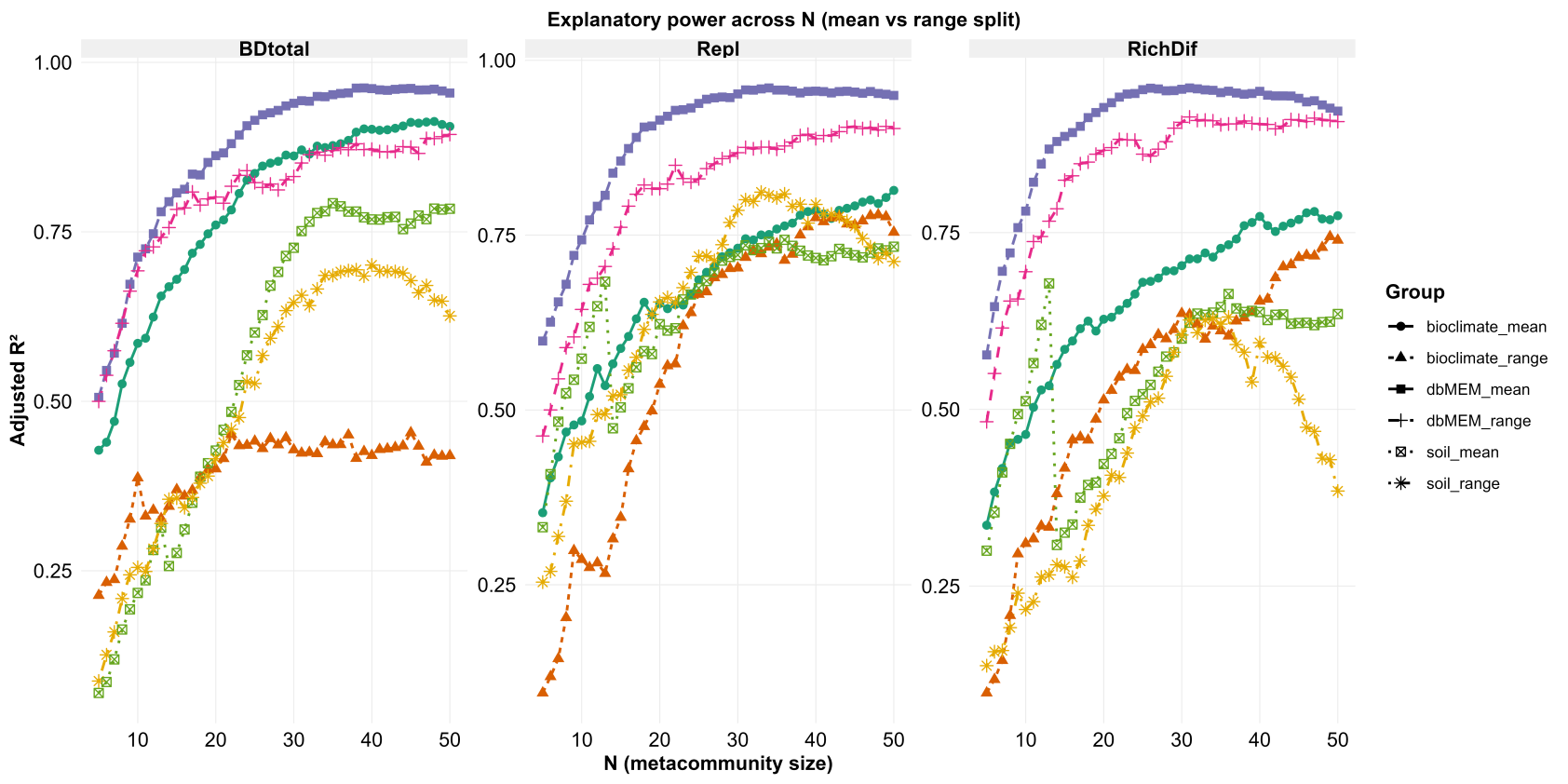


Figure S4.1 Adjusted *R*^2^ of six single-group RDA models across metacommunity size *N*, split by mean and range components within each predictor family (bioclimate_mean/range_, soil_mean/range_, dbMEM_mean/range_). Panels show BDtotal, Repl, and RichDif. Curves illustrate rapid gains and saturation with increasing *N* and reveal that dbMEM_mean_ explains the largest variance across responses, whereas soil_range_ approaches soil_mean_—and can exceed it for Repl—at intermediate scales.

The standardized coefficients from three-group RDA models further resolved which predictors consistently drove β-diversity across spatial extent (Figure S4.2). Within soil, standardized effects were led by SOC and pH, with range terms such as CEC (CEC_rng) and SOC (SOC_rng) frequently significant across *N*. In bioclimate, a compact subset—especially bio4, bio8, bio2, and bio15—showed large, scale-robust effects, with stronger magnitudes for Repl relative to RichDif. For dbMEM, several axes (MEM3, MEM4, MEM1/2/6, MEM8, and MEM18_rng) were persistently influential, underscoring the high spatial dimensionality behind β-diversity patterns. Overall, the heatmap highlights broad scale-robust significance for leading predictors in each set and aligns with the group-level ranking: spatial structure explains the largest absolute fraction, while bioclimate and soil—particularly their key variables—track both replacement and richness difference components.


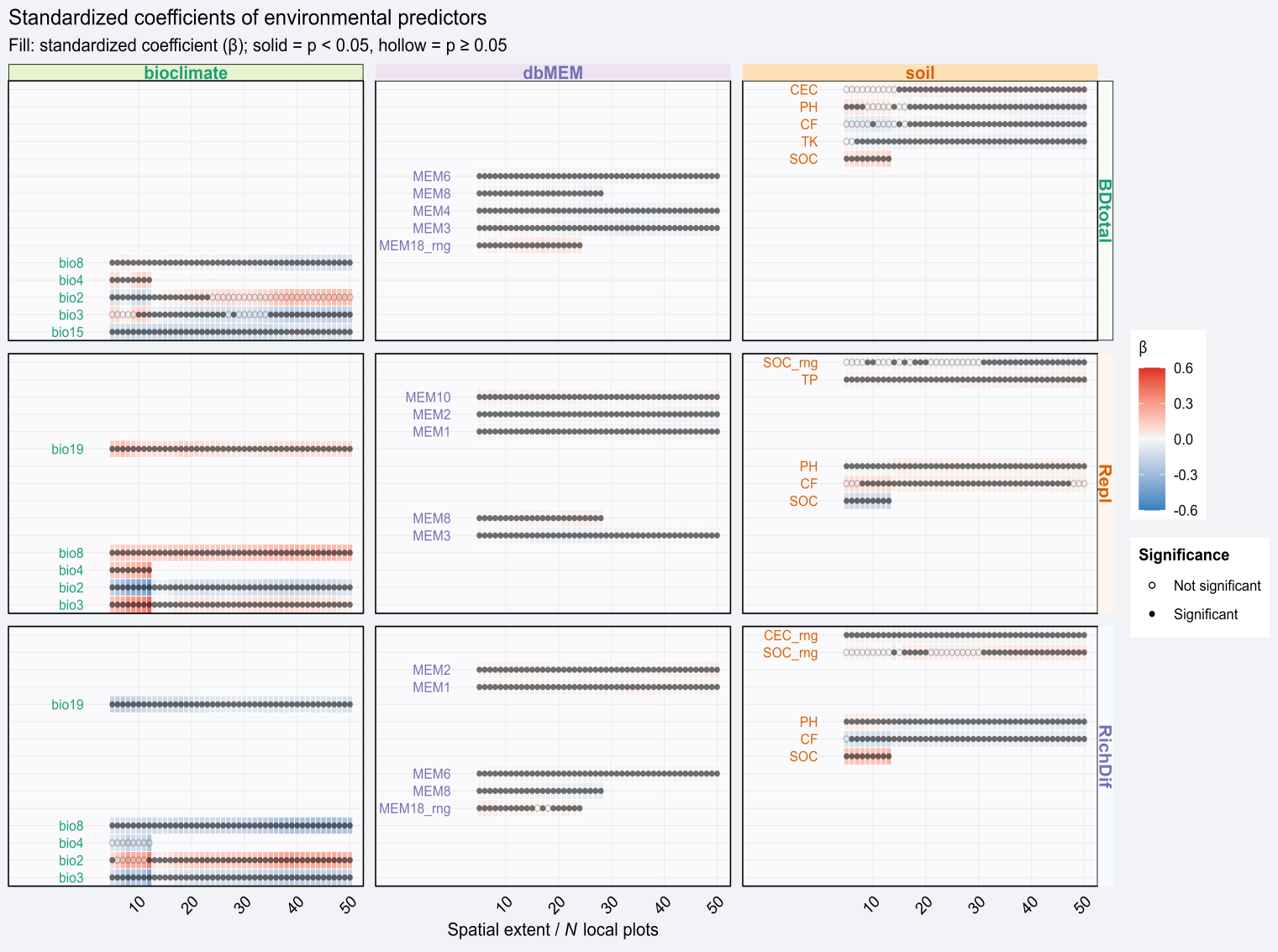


Figure S4.2 **Heatmap of standardized coefficients from three-group RDA models.** Standardized coefficients (β) from three-group RDA models across metacommunity size *N*, shown separately for bioclimate, dbMEM, and soil and for the three response metrics (BDtotal, Repl, and RichDif). Tiles depict β magnitude and sign (diverging color scale); overlaid points indicate term significance at each *N* (solid, *p* < 0.05; hollow, *p* ≥ 0.05). Rows/columns display only the highest-ranked variables within each group (by mean |β| across scales). Variable abbreviations are shown on the left of each panel refer to the Table S1 for the corresponding variable descriptions, and the Material and Methods 2.2.1.

Univariate RDA (ranked by the mean adjusted *R*^2^ across spatial extent) revealed response-specific but internally consistent patterns (Figure S4.3). For BDtotal, the Top 10 mixed bioclimate, spatial, and a single soil predictor, with bio15 ranking first, followed by MEM30_rng and TK_rng. For replacement component (Repl), the Top 10 was dominated by dbMEM axes (8/10), only bio2_rng and one soil term (TP_rng) entered the top ranks. For RichDif, the Top 10 was almost exclusively dbMEM (9/10), the only non-spatial entry was bio2_rng. Mean scale-wise significance for the Top-10 was 99.8% (BDtotal), 100% (Repl), and 94.1% (RichDif). These single-predictor results show that individual spatial axes (dbMEM) have the strongest associations with replacement **and richness differences**, whereas **BDtotal** integrates both **bioclimatic** and **spatial** signals, with only **limited soil entries** reaching top 10.

**
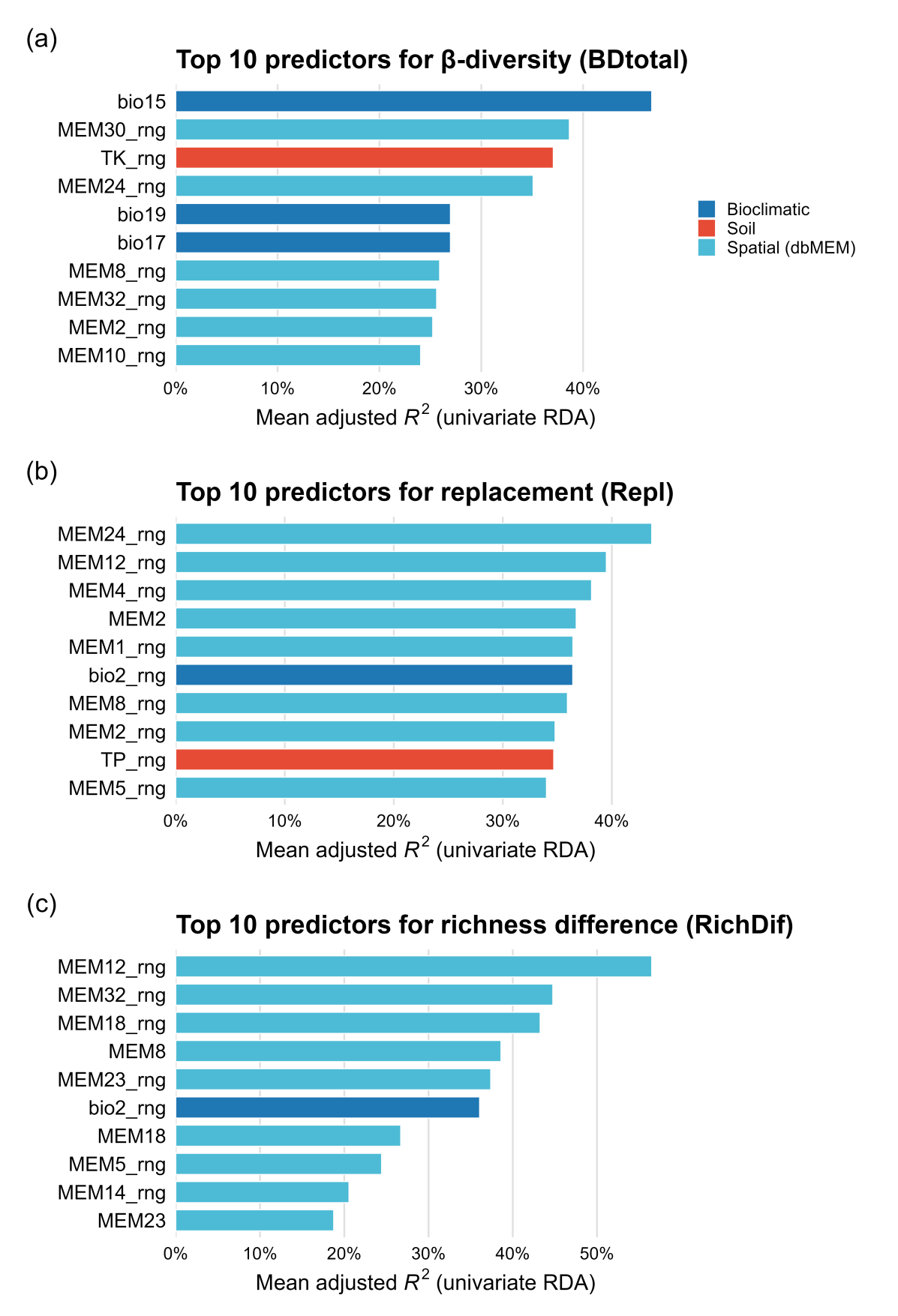
**

Figure S4.3 **Top 10 univariate predictors ranked by mean adjusted *R*^2^**. (a) total β-diversity (BDtotal), (b) replacement (Repl), and (c) richness difference component (RichDif), ranked by mean adjusted *R*^2^ across *N* = 5 – 50 local plots; bars colored by predictor family (bioclimate, soil, and dbMEM). Variable abbreviations are shown on the left of the each panel; refer to the Table S1 for the corresponding variable descriptions, and the Material and Methods 2.2.1.

**Table S1. Predictor variables used in RDA analyses.** Predictor group indicates the broad set of variables; variable family groups related variables. Summary type indicates how plot-level values were summarized for each metacommunity: Mean = average across the N local plots; Range = maximum minus minimum across the N local plots. MEM variables are distance-based Moran eigenvector map axes derived from plot coordinates and used as spatial structure predictors; lower-order MEMs generally represent broader spatial patterns, whereas higher-order selected MEMs represent finer spatial patterns.

| **Variable** | **Predictor group** | **Variable family** | **Summary type** | **Variable meaning** |
| --- | --- | --- | --- | --- |
| bio1 | Bioclimate | Temperature | Mean (average across N plots) | Annual mean temperature |
| bio1_rng | Bioclimate | Temperature | Range (max–min across N plots) | Annual mean temperature |
| bio2 | Bioclimate | Temperature | Mean (average across N plots) | Mean diurnal temperature range |
| bio2_rng | Bioclimate | Temperature | Range (max–min across N plots) | Mean diurnal temperature range |
| bio3 | Bioclimate | Temperature | Mean (average across N plots) | Isothermality |
| bio3_rng | Bioclimate | Temperature | Range (max–min across N plots) | Isothermality |
| bio4 | Bioclimate | Temperature | Mean (average across N plots) | Temperature seasonality |
| bio4_rng | Bioclimate | Temperature | Range (max–min across N plots) | Temperature seasonality |
| bio5 | Bioclimate | Temperature | Mean (average across N plots) | Maximum temperature of warmest month |
| bio5_rng | Bioclimate | Temperature | Range (max–min across N plots) | Maximum temperature of warmest month |
| bio6 | Bioclimate | Temperature | Mean (average across N plots) | Minimum temperature of coldest month |
| bio6_rng | Bioclimate | Temperature | Range (max–min across N plots) | Minimum temperature of coldest month |
| bio7 | Bioclimate | Temperature | Mean (average across N plots) | Temperature annual range |
| bio7_rng | Bioclimate | Temperature | Range (max–min across N plots) | Temperature annual range |
| bio8 | Bioclimate | Temperature | Mean (average across N plots) | Mean temperature of wettest quarter |
| bio8_rng | Bioclimate | Temperature | Range (max–min across N plots) | Mean temperature of wettest quarter |
| bio9 | Bioclimate | Temperature | Mean (average across N plots) | Mean temperature of driest quarter |
| bio9_rng | Bioclimate | Temperature | Range (max–min across N plots) | Mean temperature of driest quarter |
| bio10 | Bioclimate | Temperature | Mean (average across N plots) | Mean temperature of warmest quarter |
| bio10_rng | Bioclimate | Temperature | Range (max–min across N plots) | Mean temperature of warmest quarter |
| bio11 | Bioclimate | Temperature | Mean (average across N plots) | Mean temperature of coldest quarter |
| bio11_rng | Bioclimate | Temperature | Range (max–min across N plots) | Mean temperature of coldest quarter |
| bio12 | Bioclimate | Precipitation | Mean (average across N plots) | Annual precipitation |
| bio12_rng | Bioclimate | Precipitation | Range (max–min across N plots) | Annual precipitation |
| bio13 | Bioclimate | Precipitation | Mean (average across N plots) | Precipitation of wettest month |
| bio13_rng | Bioclimate | Precipitation | Range (max–min across N plots) | Precipitation of wettest month |
| bio14 | Bioclimate | Precipitation | Mean (average across N plots) | Precipitation of driest month |
| bio14_rng | Bioclimate | Precipitation | Range (max–min across N plots) | Precipitation of driest month |
| bio15 | Bioclimate | Precipitation | Mean (average across N plots) | Precipitation seasonality |
| bio15_rng | Bioclimate | Precipitation | Range (max–min across N plots) | Precipitation seasonality |
| bio16 | Bioclimate | Precipitation | Mean (average across N plots) | Precipitation of wettest quarter |
| bio16_rng | Bioclimate | Precipitation | Range (max–min across N plots) | Precipitation of wettest quarter |
| bio17 | Bioclimate | Precipitation | Mean (average across N plots) | Precipitation of driest quarter |
| bio17_rng | Bioclimate | Precipitation | Range (max–min across N plots) | Precipitation of driest quarter |
| bio18 | Bioclimate | Precipitation | Mean (average across N plots) | Precipitation of warmest quarter |
| bio18_rng | Bioclimate | Precipitation | Range (max–min across N plots) | Precipitation of warmest quarter |
| bio19 | Bioclimate | Precipitation | Mean (average across N plots) | Precipitation of coldest quarter |
| bio19_rng | Bioclimate | Precipitation | Range (max–min across N plots) | Precipitation of coldest quarter |
| BD | Soil | Soil physical properties | Mean (average across N plots) | Bulk density |
| BD_rng | Soil | Soil physical properties | Range (max–min across N plots) | Bulk density |
| ST | Soil | Soil physical properties | Mean (average across N plots) | Soil thickness |
| ST_rng | Soil | Soil physical properties | Range (max–min across N plots) | Soil thickness |
| CF | Soil | Soil physical properties | Mean (average across N plots) | Coarse fragments |
| CF_rng | Soil | Soil physical properties | Range (max–min across N plots) | Coarse fragments |
| PH | Soil | Soil acidity and exchange capacity | Mean (average across N plots) | Soil pH |
| PH_rng | Soil | Soil acidity and exchange capacity | Range (max–min across N plots) | Soil pH |
| TK | Soil | Soil nutrient pools | Mean (average across N plots) | Total potassium |
| TK_rng | Soil | Soil nutrient pools | Range (max–min across N plots) | Total potassium |
| TN | Soil | Soil nutrient pools | Mean (average across N plots) | Total nitrogen |
| TN_rng | Soil | Soil nutrient pools | Range (max–min across N plots) | Total nitrogen |
| TP | Soil | Soil nutrient pools | Mean (average across N plots) | Total phosphorus |
| TP_rng | Soil | Soil nutrient pools | Range (max–min across N plots) | Total phosphorus |
| SOC | Soil | Soil organic matter | Mean (average across N plots) | Soil organic carbon |
| SOC_rng | Soil | Soil organic matter | Range (max–min across N plots) | Soil organic carbon |
| CEC | Soil | Soil acidity and exchange capacity | Mean (average across N plots) | Cation exchange capacity |
| CEC_rng | Soil | Soil acidity and exchange capacity | Range (max–min across N plots) | Cation exchange capacity |
| MEM1 | Spatial structure (dbMEM) | Spatial autocorrelation axis | Mean (average across N plots) | dbMEM axis 1; orthogonal spatial predictor derived from plot coordinates |
| MEM1_rng | Spatial structure (dbMEM) | Spatial autocorrelation axis | Range (max–min across N plots) | dbMEM axis 1; orthogonal spatial predictor derived from plot coordinates |
| MEM2 | Spatial structure (dbMEM) | Spatial autocorrelation axis | Mean (average across N plots) | dbMEM axis 2; orthogonal spatial predictor derived from plot coordinates |
| MEM2_rng | Spatial structure (dbMEM) | Spatial autocorrelation axis | Range (max–min across N plots) | dbMEM axis 2; orthogonal spatial predictor derived from plot coordinates |
| MEM3 | Spatial structure (dbMEM) | Spatial autocorrelation axis | Mean (average across N plots) | dbMEM axis 3; orthogonal spatial predictor derived from plot coordinates |
| MEM3_rng | Spatial structure (dbMEM) | Spatial autocorrelation axis | Range (max–min across N plots) | dbMEM axis 3; orthogonal spatial predictor derived from plot coordinates |
| MEM4 | Spatial structure (dbMEM) | Spatial autocorrelation axis | Mean (average across N plots) | dbMEM axis 4; orthogonal spatial predictor derived from plot coordinates |
| MEM4_rng | Spatial structure (dbMEM) | Spatial autocorrelation axis | Range (max–min across N plots) | dbMEM axis 4; orthogonal spatial predictor derived from plot coordinates |
| MEM5 | Spatial structure (dbMEM) | Spatial autocorrelation axis | Mean (average across N plots) | dbMEM axis 5; orthogonal spatial predictor derived from plot coordinates |
| MEM5_rng | Spatial structure (dbMEM) | Spatial autocorrelation axis | Range (max–min across N plots) | dbMEM axis 5; orthogonal spatial predictor derived from plot coordinates |
| MEM6 | Spatial structure (dbMEM) | Spatial autocorrelation axis | Mean (average across N plots) | dbMEM axis 6; orthogonal spatial predictor derived from plot coordinates |
| MEM6_rng | Spatial structure (dbMEM) | Spatial autocorrelation axis | Range (max–min across N plots) | dbMEM axis 6; orthogonal spatial predictor derived from plot coordinates |
| MEM7 | Spatial structure (dbMEM) | Spatial autocorrelation axis | Mean (average across N plots) | dbMEM axis 7; orthogonal spatial predictor derived from plot coordinates |
| MEM7_rng | Spatial structure (dbMEM) | Spatial autocorrelation axis | Range (max–min across N plots) | dbMEM axis 7; orthogonal spatial predictor derived from plot coordinates |
| MEM8 | Spatial structure (dbMEM) | Spatial autocorrelation axis | Mean (average across N plots) | dbMEM axis 8; orthogonal spatial predictor derived from plot coordinates |
| MEM8_rng | Spatial structure (dbMEM) | Spatial autocorrelation axis | Range (max–min across N plots) | dbMEM axis 8; orthogonal spatial predictor derived from plot coordinates |
| MEM10 | Spatial structure (dbMEM) | Spatial autocorrelation axis | Mean (average across N plots) | dbMEM axis 10; orthogonal spatial predictor derived from plot coordinates |
| MEM10_rng | Spatial structure (dbMEM) | Spatial autocorrelation axis | Range (max–min across N plots) | dbMEM axis 10; orthogonal spatial predictor derived from plot coordinates |
| MEM12 | Spatial structure (dbMEM) | Spatial autocorrelation axis | Mean (average across N plots) | dbMEM axis 12; orthogonal spatial predictor derived from plot coordinates |
| MEM12_rng | Spatial structure (dbMEM) | Spatial autocorrelation axis | Range (max–min across N plots) | dbMEM axis 12; orthogonal spatial predictor derived from plot coordinates |
| MEM13 | Spatial structure (dbMEM) | Spatial autocorrelation axis | Mean (average across N plots) | dbMEM axis 13; orthogonal spatial predictor derived from plot coordinates |
| MEM13_rng | Spatial structure (dbMEM) | Spatial autocorrelation axis | Range (max–min across N plots) | dbMEM axis 13; orthogonal spatial predictor derived from plot coordinates |
| MEM14 | Spatial structure (dbMEM) | Spatial autocorrelation axis | Mean (average across N plots) | dbMEM axis 14; orthogonal spatial predictor derived from plot coordinates |
| MEM14_rng | Spatial structure (dbMEM) | Spatial autocorrelation axis | Range (max–min across N plots) | dbMEM axis 14; orthogonal spatial predictor derived from plot coordinates |
| MEM18 | Spatial structure (dbMEM) | Spatial autocorrelation axis | Mean (average across N plots) | dbMEM axis 18; orthogonal spatial predictor derived from plot coordinates |
| MEM18_rng | Spatial structure (dbMEM) | Spatial autocorrelation axis | Range (max–min across N plots) | dbMEM axis 18; orthogonal spatial predictor derived from plot coordinates |
| MEM21 | Spatial structure (dbMEM) | Spatial autocorrelation axis | Mean (average across N plots) | dbMEM axis 21; orthogonal spatial predictor derived from plot coordinates |
| MEM21_rng | Spatial structure (dbMEM) | Spatial autocorrelation axis | Range (max–min across N plots) | dbMEM axis 21; orthogonal spatial predictor derived from plot coordinates |
| MEM23 | Spatial structure (dbMEM) | Spatial autocorrelation axis | Mean (average across N plots) | dbMEM axis 23; orthogonal spatial predictor derived from plot coordinates |
| MEM23_rng | Spatial structure (dbMEM) | Spatial autocorrelation axis | Range (max–min across N plots) | dbMEM axis 23; orthogonal spatial predictor derived from plot coordinates |
| MEM24 | Spatial structure (dbMEM) | Spatial autocorrelation axis | Mean (average across N plots) | dbMEM axis 24; orthogonal spatial predictor derived from plot coordinates |
| MEM24_rng | Spatial structure (dbMEM) | Spatial autocorrelation axis | Range (max–min across N plots) | dbMEM axis 24; orthogonal spatial predictor derived from plot coordinates |
| MEM30 | Spatial structure (dbMEM) | Spatial autocorrelation axis | Mean (average across N plots) | dbMEM axis 30; orthogonal spatial predictor derived from plot coordinates |
| MEM30_rng | Spatial structure (dbMEM) | Spatial autocorrelation axis | Range (max–min across N plots) | dbMEM axis 30; orthogonal spatial predictor derived from plot coordinates |
| MEM32 | Spatial structure (dbMEM) | Spatial autocorrelation axis | Mean (average across N plots) | dbMEM axis 32; orthogonal spatial predictor derived from plot coordinates |
| MEM32_rng | Spatial structure (dbMEM) | Spatial autocorrelation axis | Range (max–min across N plots) | dbMEM axis 32; orthogonal spatial predictor derived from plot coordinates |
